# Effects of a Mobile and Web App (Thought Spot) on Mental Health Help-Seeking Among College and University Students: Randomized Controlled Trial

**DOI:** 10.2196/20790

**Published:** 2020-10-30

**Authors:** David Wiljer, Jenny Shi, Brian Lo, Marcos Sanches, Elisa Hollenberg, Andrew Johnson, Alexxa Abi-Jaoudé, Gloria Chaim, Kristin Cleverley, Joanna Henderson, Wanrudee Isaranuwatchai, Andrea Levinson, Janine Robb, Howard W Wong, Aristotle Voineskos

**Affiliations:** 1 UHN Digital University Health Network Toronto, ON Canada; 2 Office of Education Centre for Addiction and Mental Health Toronto, ON Canada; 3 Institute of Health Policy, Management and Evaluation University of Toronto Toronto, ON Canada; 4 Department of Psychiatry Faculty of Medicine University of Toronto Toronto, ON Canada; 5 Information Management Group Centre for Addiction and Mental Health Toronto, ON Canada; 6 Krembil Centre for Neuroinformatics Centre for Addiction and Mental Health Toronto, ON Canada; 7 Margaret and Wallace McCain Centre for Child, Youth & Family Mental Health Centre for Addiction and Mental Health Toronto, ON Canada; 8 Lawrence S Bloomberg Faculty of Nursing University of Toronto Toronto, ON Canada; 9 Centre for Excellence in Economic Analysis Research St Michael’s Hospital Toronto, ON Canada; 10 Health and Wellness University of Toronto Toronto, ON Canada; 11 Slaight Family Centre for Youth in Transition Centre for Addiction and Mental Health Toronto, ON Canada

**Keywords:** crowdsourcing, help-seeking behavior, mental health, mobile applications, randomized controlled trial, school mental health services, social support, young adult

## Abstract

**Background:**

Mental health disorders are the most prevalent health issues among postsecondary students, yet few solutions to this emerging crisis exist. While mobile health technologies are touted as promising solutions for the unmet mental health needs of these students, the efficacy of these tools remains unclear. In response to these gaps, this study evaluates Thought Spot, a mobile and web app created through participatory design research.

**Objective:**

The goal of the research is to examine the impact of Thought Spot on mental health and wellness help-seeking intentions, behaviors, attitudes, self-stigma, and self-efficacy among postsecondary students in Canada.

**Methods:**

A 2-armed randomized controlled trial involving students from three postsecondary institutions was conducted. Students were eligible if they were aged 17 to 29 years, enrolled in full-time or part-time studies, functionally competent in English, and had access to a compatible digital device. The usual care group received a mental health services information pamphlet. The intervention group received the Thought Spot app on their digital device. Thought Spot is a standalone app that allows users to add, review, and search crowdsourced information about nearby mental health and wellness services. Users can also track their mood on the app. Outcomes were self-assessed through questionnaires collected at baseline and 3 and 6 months. The primary outcome was change in formal help-seeking intentions from baseline to 6 months, measured by the General Help-Seeking Questionnaire. A mixed-effects model was used to compare the impact of usual care and intervention on the primary outcome (formal help-seeking intentions). Secondary outcomes included changes in informal help-seeking intentions and help-seeking behaviors, help-seeking attitudes, self-stigma, and self-efficacy.

**Results:**

A total of 481 students were randomized into two groups: 240 to usual care, and 241 to the intervention group. There were no significant differences in help-seeking intentions between the usual care and intervention groups over 6 months (*F*_2,877_=0.85; *P*=.43, *f*=0.04). Both groups demonstrated similar increases in formal help-seeking intentions at 3 and 6 months (*F*_2,877_=23.52; *P*<.001, *f*=0.21). Compared with males, females sought more help from formal resources (OR 1.86; 95% CI 1.22 to 2.83, *P*=.001). Females were less likely to seek help from informal sources than males (OR 0.80; 95% CI 0.22 to 0.73, *P*<.001).

**Conclusions:**

Prompting postsecondary students about mental health and help-seeking appears to increase help-seeking intentions. mHealth interventions may be as effective as information pamphlets in increasing formal help-seeking but may confer a small advantage in driving help-seeking from informal sources. Although there is enthusiasm, developers and health policy experts should exercise caution and thoroughly evaluate these types of digital tools. Future studies should explore the cost-effectiveness of digital interventions and develop strategies for improving their efficacy.

**Trial Registration:**

ClinicalTrials.gov NCT03412461; https://clinicaltrials.gov/ct2/show/NCT03412461

**International Registered Report Identifier (IRRID):**

RR2-10.2196/resprot.6446

## Introduction

Mental health disorders among postsecondary students are a global public health concern [1-4]. Youth aged 16 to 29 years face many challenges in the transition from childhood to adulthood, and 70% of all mental health conditions have their onset during this period [5-8]. Postsecondary education is an experience that can pose many challenges for transition-aged youth due to social, financial, and academic stressors [3,9-11]. Over the last 10 years, rates of mental health disorders among postsecondary students have increased [12]. Yet 35% of youth who are experiencing mental health problems do not seek formal help (eg, clinical services, health professionals) [13] or informal help (eg, friends and family, religious leaders, self-help) [13] due to barriers such as perceived stigma, difficulty expressing their concerns, difficulty accessing help, and a preference for self-reliance [14,15]. Amid the “campus mental health crisis” [1], there is a desperate need for interventions that facilitate help-seeking and access to mental health and wellness services [16].

The proliferation of mobile devices and their ubiquity in the lives of transition-aged youth has encouraged developers, postsecondary institutions, and health care organizations to focus their efforts on online and mobile health (mHealth) interventions that address the mental health challenges faced by this population [17-20]. The evidence to support mHealth solutions has not been established, but they remain a major hope for transition-aged youth, given the extraordinary challenges and costs of addressing the mental health needs of postsecondary students [21-23]. To date, assessments of most mHealth interventions have been limited to case studies, pilot studies, and randomized controlled trials (RCTs) with small sample sizes [22]. Few studies involve user-centered design processes or end users as co-creators, evidence-based strategies that help mHealth tools achieve sustained engagement, effectiveness, and behavior change [24].

**Figure 1:**
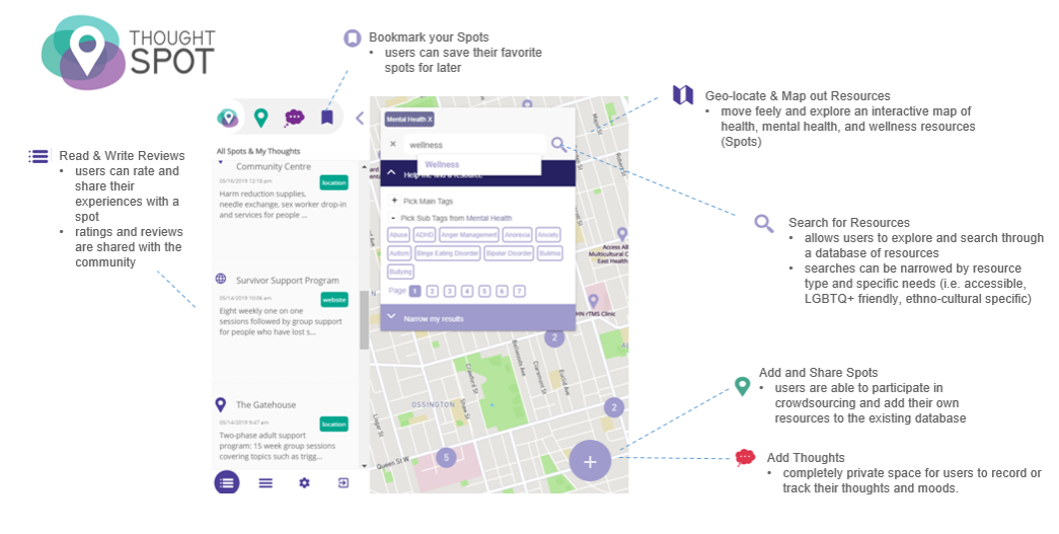
Features on the Thought Spot mobile app and online platform, designed by students for students to allow users to find and share health, mental health, and wellness resources (spots) using a map-based database of crowdsourced resources and a self-contained search feature.

Applying principles from participatory action and participatory design research [25,26], the research team produced Thought Spot, an mHealth intervention co-created with transition-aged youth that aims to improve help-seeking behavior related to mental health services for postsecondary settings [27,28]. Thought Spot serves as a map-based database (via a mobile app and website) that allows users to search and geolocate health, mental health, and wellness resources (Figure 1). Additional features of the app include mood and thought tracking, reviews about nearby resources and services, and the ability to bookmark searched information. Thought Spot users can also participate in crowdsourcing by adding new resources and writing reviews. Accordingly, the main objective of this RCT was to assess the impact of Thought Spot on intentions to seek formal help. The secondary objective was to examine the impact on intentions to seek help from informal sources and on help-seeking behaviors, help-seeking attitudes, self-stigma, and self-efficacy [17]. The research team hypothesized that Thought Spot would be superior to school-specific mental health services information pamphlets in increasing formal help-seeking intentions.

## Methods

### Study Design

The team conducted a 2-armed RCT using participants who were students at three postsecondary institutions in the Greater Toronto Area (University of Toronto, Ryerson University, and George Brown College). The study staff and biostatistician were blinded throughout the study. The protocol was approved by the research ethics boards of each participating postsecondary institution and the Centre for Addiction and Mental Health and has been previously published (Multimedia Appendix 1) [27]. The trial was registered at ClinicalTrials.gov [NCT03412461]. Digital informed consent was obtained from each participant through Research Electronic Data Capture (REDCap) before participants could be included in the trial [29].

### Recruitment

Participants were recruited using the most effective methods identified by two focus groups and the Thought Spot youth advisory committee during earlier stages of the project [28]. Participants were recruited through institutional and student-related listservs, bulletin boards, websites, social media, and class presentations.

**Figure 2:**
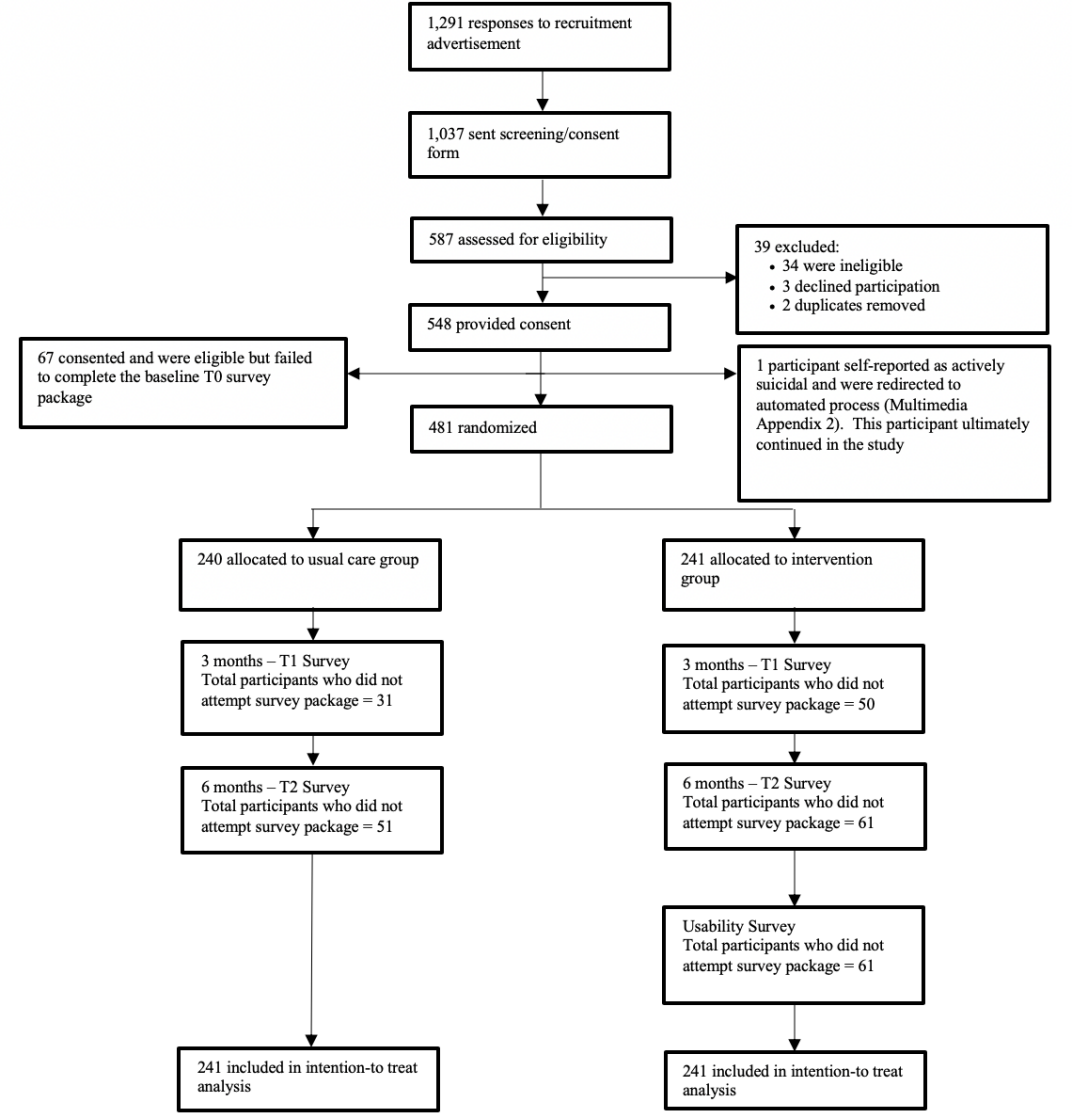
Consolidated System of Reporting Trials flow diagram detailing the order of screening, randomization, and follow-up procedures.

Full-time and part-time students aged 17 to 29 years who were enrolled at any of the three postsecondary institutions, who were functionally competent in English, and who had access to a digital device compatible with the intervention were eligible to participate in the study. Active suicidality was the sole exclusion criterion, but no participants met this criterion during the screening process (Figure 2; Multimedia Appendix 2). Participants also did not have knowledge of or access to Thought Spot prior to participating in the study.

### Randomization and Treatment

Participants were randomly assigned using a 1:1 allocation ratio to either receive access to Thought Spot or to receive a school-specific mental health services information pamphlet. Randomization and allocation was performed using REDCap, a secure, browser-based, electronic data capture system [29]. A randomization module within REDCap provided computer-generated random allocation of participants in blocks of 10. All participants were enrolled by EH. All authors were blind to the allocation except for JS, who oversaw the randomization process. REDCap was configured to collect data through online questionnaires at baseline, 3 months and 6 months. We asked participants in both groups to use their intervention (Thought Spot or pamphlet) as needed over 6 months. Intervention group participants received detailed instructions via email on how to download and access the Thought Spot app on their personal electronic devices. Participants were also sent two study reminders, at 6 weeks and 18 weeks, in anticipation of the 3-month and 6-month questionnaires. At the end of the study, participants in the intervention group completed an adapted version of the Usefulness, Satisfaction, and Ease of Use questionnaire [30] and were invited to participate in qualitative interviews to evaluate Thought Spot’s usability and user experience. Full details on the randomization process and interventions are provided in the trial protocol in Multimedia Appendix 1. There were no changes to methods or major changes to the features and functionalities of the app. The content on the app changed by design because participants could add their own crowdsourced information about mental health resources and services during the trial. There were multiple bug fixes to address usability issues, such as malfunctioning buttons, slowness of search features, and log-in credentials not being saved. There were no major downtimes during the study period. Participants were provided with a small honorarium (up to CAD $40 [USD $30.08]) for participating in the study.

### Assessment Measures

The primary outcome was a change in formal help-seeking intentions from baseline to 6 months measured by the General Help-Seeking Questionnaire (GHSQ) [31]. This scale comprises ten 7-point Likert scale questions that ask participants how likely they are to seek help from different sources, including friends, family members, mental health professionals, and helplines [31]. The formal help-seeking intentions scores were calculated by summing the scores of the 3 questions measuring formal help-seeking intentions [31]. Secondary outcomes of the study included changes in informal help-seeking intentions and help-seeking behaviors, help-seeking attitudes, self-stigma, and self-efficacy, measured, respectively, by the Actual Help-Seeking Questionnaire (AHSQ) [31], Attitudes Toward Seeking Professional Psychological Help Scale–Short Form (ATSPPH-SF) [32], Self-Stigma of Seeking Help Scale (SSOSH) [33], and the Youth Efficacy/Empowerment Scale–Mental Health (YES-MH) [34].

### Statistical Analysis

Statistical analysis was performed by a biostatistician (MS) who was blinded to randomization. To determine the required sample size, power calculations were conducted using the primary outcome, the average formal help-seeking score on the GHSQ [27]. Monte Carlo simulations were conducted in SAS 9.4 for Windows (SAS Institute Inc) using the means, standard deviations, and within-subject associations reported in previous research using the GHSQ [35,36]. Monte Carlo simulations were chosen because our hypothesis required a model that accounted for dropouts, random effects, and the specific use of linear contrast to compare pre with post. The calculation accounted for a 40% dropout rate and assumed the use of a mixed-effects model to test the primary hypothesis at a significance level of .05 (2-tailed). A sample of 472 participants (236 per arm) was found to provide 80% power to detect a small effect size (Cohen *d*=0.25), which is equivalent to a 15% difference in change in GHSQ score from baseline to 6 months.

Univariate analyses were conducted to describe groups at baseline (Table 1) and compare completers and noncompleters of the questionnaires. Completers were defined as participants who finished the survey at all 3 points of the study [27]. To evaluate the primary hypothesis, the research team used mixed-effects models using the intention-to-treat method, where time, group, and gender were entered as fixed effects and participants were entered as random effects. A linear contrast of estimated marginal means from the fixed-effect interaction between time and group was conducted to compare changes from baseline to 6 months across groups. Missing values were handled by maximum likelihood estimation, which is able to incorporate all available information in the data under the missing at random assumptions [37]. The team conducted sensitivity analyses, in which we added model variables found to have significant bivariate associations with dropouts, defined as participants who were randomized but who did not complete all 3 surveys, at a significance level of α=.20.

**Table 1:** Baseline characteristics of participants who underwent randomization^a^.

Characteristics		Control (n=240)	Treatment (n=241)
**Gender, n (%)**			
	Female	188 (78.3)	190 (78.6)
	Male	47 (19.6)	44 (18.9)
	Nonbinary	5 (2.1)	7 (2.5)
Age in years, mean (SD)		23.2 (3.1)	22.9 (3.4)
**Student status, n (%)**			
	Domestic student	207 (86.3)	207 (85.9)
	International student	31 (12.9)	33 (13.7)
	Don’t know	2 (0.8)	1 (0.4)
**Education level, n (%)**			
	High school diploma	144 (60.0)	140 (58.1)
	College diploma	23 (9.6)	9 (3.7)
	Bachelor’s degree	59 (24.6)	74 (30.7)
	Master’s degree	11 (4.6)	9 (4.2)
	Doctoral degree	0	0
	Other	3 (1.3)	9 (3.7)
**Type of postsecondary institution^b^, n (%)**			
	College	110 (45.8)	114 (47.3)
	University	130 (54.2)	125 (51.9)
	Did not answer	0	2 (0.8)
**Experience with mental health or substance use concerns, n (%)**			
	Yes	168 (70.0)	172 (70.8)
	No	55 (23.0)	63 (26.1)
	Don’t know	16 (6.7)	6 (4.6)
	Did not answer	1 (0.4)	0
GHSQ^c^ formal sources, mean (SD)		8.6 (3.9)	8.3 (4.1)
GHSQ informal sources, mean (SD)		36.6 (8.5)	36.1 (9.4)

^a^Percentages may not total 100 due to rounding. There was no significant difference between the trial groups.

^b^Postsecondary institution was missing for two participants.

^c^GHSQ: General Help-Seeking Questionnaire.

Similar models were conducted to explore the secondary outcomes of this study except for help-seeking behavior. This outcome was measured using the binary-scaled AHSQ and analyzed using a mixed binomial logistic regression model. The *P* values for the secondary analysis were not adjusted for multiple comparison. In addition to the prespecified analysis plan, the research team conducted an exploratory per-protocol analysis of the primary and secondary outcomes. Participants who logged on to Thought Spot more than once during the study were considered compliant.

For *F* tests, the study report standardized effect sizes using Cohen *f* [38], reported as *f*, which is calculated by the R package sjstats (R Foundation for Statistical Computing) [39]. For contrasts that represent simple changes or difference in changes, Cohen *d* was used, which is reported as *d*. The respective *d* values are calculated using estimated marginal means and two formulas described by Morris and DeShon [40]. The biostatistician applied their recommended formula for repeated-measures design that focuses on change within a person relative to the variability of change scores and another formula used for independent groups pretest/posttest designs (Multimedia Appendix 3) [40].

## Results

### Recruitment and Participant Characteristics

From March 2018 to January 2019, 481 participants were randomized from three Canadian postsecondary institutions into a 6-month trial on a rolling basis. Of these participants, 240 were assigned to the control group and 241 were assigned to the intervention group (Figure 2). The trial ended after all participants received their 6-month follow-up on June 30, 2019. Table 1 shows a comparison of study groups at baseline. Prior experience with mental health concerns was reported by 70.7% (340/481) of participants, and 61.3% (294/480) of participants reported having experienced suicidal ideation in their lifetime. There were no reported adverse events or harms during the trial.

### Primary Outcome: Change in Formal Help-Seeking Intentions

The mixed-effects model found a significant time effect (*F*_2,877_=23.52; *P*<.001, *f*=0.21) but not a significant group-by-time interaction (*F*_2,877_=0.85; *P*=.43, *f*=0.04). Linear contrasts exploring the change in formal help-seeking intentions between baseline and 6 months did not find significant differences between the intervention and control group (estimate mean difference in change 0.39; t_894_=1.05; 95% CI –0.34 to 1.12; *P*=.29; *d*=0.09). Both the control group (estimated mean change 1.32; 95% CI 0.81 to 1.84, *d*=0.33) and the intervention group (estimated mean difference 0.93, 95% CI 0.41 to 1.45, *d*=0.24) showed a significant increase in the intention to seek formal sources of help. A sensitivity analysis revealed that group-by-time interaction was not significant after controlling for factors that were associated with dropout (*F*_2,145_=1.18, *P*=.31, *f*=0.11). There was also no difference in changes between groups (estimate 1.35, 95% CI –0.51 to 3.21, *d*=0.32).

### Secondary Outcomes

An analysis of secondary outcomes found no significant group-by-time interactions for help-seeking intentions from informal sources (GHSQ), help-seeking behavior from formal sources (AHSQ), help-seeking attitudes toward professional help (ATSPPH), self-efficacy (SSOSH) and self-stigma (YES-MH; Table 2). However, some evidence of a group-by-time interaction was found for help-seeking from informal sources (Wald χ^2^=4.5, *P*=.11), but it is not significant (Table 3). Linear contrasts of the interaction indicated a decrease in help-seeking behavior related to informal sources in the control group, whereas an increase from 3 months to 6 months was found for the intervention group (odds ratio [OR] 0.86; 95% CI 0.71 to 1.02, *P*=.09), but it did not meet the significance threshold (Multimedia Appendix 4).

**Table 2:** Main results from mixed models.

Participant questionnaires		Linear contrast^a^			Group-by-time interaction^b^		
		Estimate^a^	95% CI	*P* value	*F* statistic	DF^c^	*P* value
**GHSQ^d^ (intention to treat)**							
	Formal resources	0.39	–0.34 to 1.12	.30	0.85	2/877	.43
	Informal resources	0.36	–1.21 to 1.92	.65	0.70	2/811	.50
GHSQ (per-protocol) – formal resources		0.18	–0.62 to 0.98	.66	0.14	2/757	.87
GHSQ (dropout)^b^ – formal resources		1.35	–0.51 to 3.21	.15	1.18	2/145	.31
GHSQ (controlled for site)^e^ – formal resources		0.39	0.34 to 1.13	.29	0.85	2/877	.43
ATSPPH^f^		–0.15	–0.91 to 0.61	.70	1.39	2/850	.25
SSOSH^g^		0.08	–1.13 to 1.28	.90	0.03	2/876	.97
YES-MH^h^: self		–0.09	–0.73 to 0.55	.79	0.12	2/801	.89
YES-MH: service		0.47	–0.40 to 1.34	.29	0.60	2/823	.55
YES-MH: system		0.62	–0.26 to 1.50	.17	1.31	2/818	.27
YES-MH: total		0.16	–1.52 to 1.83	.85	0.08	2/738	.92

^a^The linear contrast tests the change from baseline to end of the trial across groups (primary outcome); (6 months – baseline) in control minus intervention. Positive values indicate larger increases in the intervention group.

^b^The group-by-time interaction tests for any difference in the group trajectories (not primary outcome). Sensitivity analysis controls for all variables at α= .20. These variables are listed in Multimedia Appendix 7.

^c^DF: degree of freedom.

^d^GHSQ: General Help-Seeking Questionnaire.

^e^An additional sensitivity analysis controlling for site as fixed effects was conducted, but site was not significant and results were similar to those for the main model.

^f^ATSPPH: Attitudes Toward Seeking Professional Psychological Help Scale.

^g^SSOSH: Self-Stigma of Seeking Help Scale.

^h^YES-MH: Youth Efficacy/Empowerment Scale–Mental Health.

**Table 3:** Main results from binomial models.

Participant questionnaires	Linear contrast^a^			Group-by-time interaction^b^		
	Odds ratio	95% CI	*P* value	Wald χ^2^	DF^c^	*P* value
AHSQ^d^ – formal resources	0.80	0.48-1.34	.39	1.3	2	.53
AHSQ – informal resources	0.86	0.71-1.02	.09	4.5	2	.11

^a^In a binomial model, the contrast is the ratio of odds ratios. A ratio lower than 1 indicates a larger increase in the probability of positive answers in the intervention group.

^b^The statistic used in binomial models is the chi-square statistic.

^c^DF: degree of freedom.

^d^AHSQ: Actual Help-Seeking Questionnaire.

The sex effect was significant for help-seeking behavior related to formal and informal sources, help-seeking attitudes toward professional help, and self-efficacy (Multimedia Appendix 5 and 6). Compared with males, females exhibited higher formal AHSQ scores (OR 1.86; 95% CI 1.22 to 2.83, *P*=.001). Females also exhibited significantly lower AHSQ informal scores than males (OR 0.80; 95% CI 0.22 to 0.73, *P*<.001). Similarly, for attitudes toward health care professionals, females had significantly lower ATSPPH scores than males (estimate 0.80; 95% CI 0.22 to 1.38, *P*=.003).

### Per-Protocol Analysis

Of the participants in the intervention group, 70.1% (169/241) met the definition of compliance. In comparing these participants to the control group, the per-protocol analysis led to the same conclusion for all primary and secondary outcomes with nonsignificant linear contrast (difference in change 0.18, t_711_=0.439, *P*=.66, *d*=0.03). In the intervention group, compliance significantly moderated the GHSQ trajectory (*P*=.006, *F*=0.15), with compliant participants increasing their help-seeking more than noncompliant participants. This change mostly happened between baseline to 3 months.

### App Use Data

Of the 241 people randomized to the intervention group, 168 visited Thought Spot, resulting in 3696 clicks recorded between March 2018 and June 2019. Overall, users viewed 190 Spots, conducted 293 searches, and created 74 Thoughts. Spots are locations of mental health and wellness resources. Thoughts are users’ personal journal entries on the app. Details of the user data will be published independently of these findings.

## Discussion

### Principal Findings

In the analysis of our primary outcome, there were no significant differences in the formal help-seeking intentions of postsecondary students between control and intervention groups. However, both groups experienced a similar increase in formal help-seeking intentions during the 6-month study period, as assessed by the GHSQ. These findings suggest that prompting youth about mental health, regardless of the delivery method (eg, information pamphlet or mHealth intervention), may increase help-seeking intentions. Our results are consistent with findings from previous RCTs involving online mental health services, in which the interventions did not lead to significant differences in formal help-seeking compared with an active control group [41,42]. However, the similar increase in help-seeking in both arms observed in our study supports the emerging discussion in that these technologies may have a “role as another option in their toolkit” when in need of mental health and wellness resources [41].

Analyses of secondary outcomes revealed no significant group-by-time interactions for help-seeking intentions from informal sources, attitudes toward seeking professional help, self-efficacy, or self-stigma. Although not significant, there is some evidence suggesting a small increase in help-seeking behavior related to informal sources between 3 and 6 months in the intervention group, whereas a small decrease was seen in the control group. This difference contrasts with findings from previous studies of help-seeking interventions, which reported no effect on informal help-seeking [42]. Although the attitudes and intentions regarding informal help-seeking did not differ between groups, the sustained increase in informal help-seeking behavior observed between 3 and 6 months in the intervention group may indicate that Thought Spot is effective in converting intention into action [43]. The difference may also be due to previously reported advantages of online interventions over typical information pamphlets and formal sources of help, such as being anonymous, less stigmatizing, easier to access and use, and more trustworthy [41,44-49]. Nonetheless, these findings must be contextualized using data from qualitative interviews, usability data, and use data collected throughout the RCT.

When looking at the differences between AHSQ scores, the results suggest that females tend to seek help from formal sources more than males. However, when looking at the informal AHSQ scores, males sought more help from informal sources than females. This observation complements the existing literature on gender differences in transition-aged youths’ help-seeking behaviors. Based on a cross-section survey study, Findlay and Sunderland [50] found that females reported contacting formal and informal resources more than males. However, the data from this RCT show that there may be a difference in preference between formal and informal resources between genders. Another interesting finding was related to the help-seeking behavior for the nonbinary gender group. While not significant due to the small sample size, the considerable odds ratio for AHSQ formal scores suggest there may be a small effect where participants in the nonbinary gender group have higher help-seeking behavior than males.

The per-protocol analysis indicated that compliant participants increased their help-seeking more than noncompliant participants, which suggests that repeated visits may contribute to changes in help-seeking intentions. While these exploratory findings should be approached with caution, it supports the emerging interest on the impact of repeated app use on study outcomes [51]. The app evaluation framework, developed by the American Psychiatric Association, highlights the importance of user engagement in the evaluation of apps for clinical use [52]. However, while use of the app is required to enable the expected benefits, the significance of the repeated use in the context of help-seeking and whether use can be representative as meeting their needs remains unclear. As such, this finding warrants further investigation into the use data and the experiences of users.

### Comparison With Prior Work

One of the noteworthy strengths of our trial is its standing as one of the few RCTs with large sample sizes that evaluate the effectiveness of mHealth interventions for help-seeking among youth in postsecondary settings [41]. The research team saw higher than predicted retention rates of at least 73% at both 3 and 6 months. The baseline characteristics between the control and intervention groups were balanced, and the research team controlled for variables associated with the outcome (eg, GHSQ). Another strength of this study is the use of participatory action research and co-creation methodology to engage transition-aged youth throughout the development and execution of the trial [53]. This methodology has been shown to increase participation in mental health care, better address youth concerns, and produce more relevant results [54,55]. The research team was successful in engaging youth across three postsecondary institutions in a longitudinal mental health study. Given the many barriers that make meaningful youth engagement a challenge [56], obtaining 1291 individual responses during our recruitment is notable in its magnitude, especially given the short time frame (relative to other mental health trials). The high level of engagement in the study suggests that our commitment to co-creating solutions resonates with the postsecondary student population [28]. It may also reflect students’ enthusiasm for mental health solutions and mHealth-related research, suggesting a need for continued engagement with this population at a time when mental health concerns and suicide rates among youth continue to rise at postsecondary institutions [57,58].

### Limitations

The study had some limitations. Group assignment could not be blinded for participants. There were also software bugs that led to an inconsistent app environment and usability issues during the trial. These issues could have affected the level of user engagement and compliance with the intervention and ultimately the effectiveness of Thought Spot because some participants had difficulty accessing key functions of the app during certain points of the trial.

There was also a number of participants who did not complete the 3-month survey packages. There were no software bugs or reported issues that could have led to the drop in participation and the reason for this observation is unknown.

Additionally, the effect size for the change in informal help-seeking behavior was small and was noted only in the 3- to 6-month period. Similarly, due to sample size restrictions, we were unable to compare the impact of Thought Spot among international and domestic students. Given the unique barriers associated with attending college in an unfamiliar location [59], future investigations should explore whether these apps can facilitate help-seeking for international students. Further investigation and a longer follow-up period are required to evaluate the sustained impact of mHealth solutions on informal help-seeking. Finally, the development of Thought Spot and the findings from this trial are based on the unique experiences of transition-aged youth enrolled in Canadian postsecondary institutions and may not be generalizable to youth outside Canadian academic settings.

### Conclusions

In summary, there were no significant differences in formal help-seeking intentions between the control and intervention groups. Female participants sought help from formal resources more often than males, whereas males were more likely to seek help from informal sources than females. There was some evidence of a small increase in informal help-seeking behavior between 3 and 6 months in the intervention group. Both groups experienced a similar increase in formal help-seeking intentions over 6 months. These findings suggest a need to further explore the effectiveness of mHealth technologies in supporting the mental health help-seeking needs of transition-aged youth. It is increasingly important as a next step to compare the cost-effectiveness of Thought Spot and information pamphlets for understanding the feasibility and sustainability of mHealth tools compared with existing strategies [60].

## Appendix

Multimedia Appendix 1 Thought Spot randomized controlled trial protocol and statistical analysis plan.

Multimedia Appendix 2 Automated process for participants self-reporting active suicidality.

Multimedia Appendix 3 Formulas to calculate effect sizes for repeated measures and pretest/posttest research designs.

Multimedia Appendix 4 Group-by-time interaction for help-seeking behavior from informal sources.

Multimedia Appendix 5 Gender effects for help-seeking behaviors, intentions, and attitudes toward professional help and self-efficacy.

Multimedia Appendix 6 Gender effects for help-seeking behaviors, intentions, and attitudes toward professional help and self-efficacy when comparing females and nonbinary to males.

Multimedia Appendix 7 Control variables or interactions that were added to the dropout model aside from the baseline General Help Seeking Questionnaire and sex.

Multimedia Appendix 8 CONSORT-EHEALTH checklist (V 1.6.1).

## Abbreviations

AHSQ: Actual Help-Seeking Questionnaire
ATSPPH-SF: Attitudes Toward Seeking Professional Psychological Help Scale–Short Form
CAMH: Centre for Addiction and Mental Health
GHSQ: General Help-Seeking Questionnaire
mHealth: mobile health
OR: odds ratio
RCT: randomized controlled trial
REDCap: Research Electronic Data Capture
SSOSH: Self-Stigma of Seeking Help Scale
YES-MH: Youth Efficacy/Empowerment Scale–Mental Health
