# Supplementary material for: Effects of a Mobile and Web App (Thought Spot) on Mental Health Help-Seeking Among College and University Students: Randomized Controlled Trial
Source: J Med Internet Res. 2020 Oct 30;22(10):e20790. doi: 10.2196/20790 (PMC7665949; doi:10.2196/20790)
Supplement: Multimedia Appendix 1 [file jmir_v22i10e20790_app1.pdf]

Thought Spot RCT Protocol

TITLE: **Bridging the gap – Tools for finding health, mental health and wellness resources for university and college students**

PRINCIPAL INVESTIGATOR:	Dr. David Wiljer
INVESTIGATOR CONTACT:	Dr. David Wiljer<br>University Health Network<br>190 Elizabeth Street<br>R. Fraser Elliott Building RFE 3S-411<br>Toronto, ON M5G 2C4<br>T: 416-340-6322<br>Fax: 416-340-5015<br>david.wiljer@uhn.ca
CO- INVESTIGATORS:	Andrew Johnson<br>Dr. Aristotle Voineskos<br>Dr. Andrea Levinson<br>Dr. Joanna Henderson<br>Gloria Chaim<br>Alexxa Abi-Jaoude<br>Dr. Kristin Cleverley<br>Sarah Sharpe<br>Tim Tripp

**SOURCE OF SUPPORT:**	Canadian Institutes of Health Research (CIHR)<br>Centre for Addiction and Mental Health (CAMH)

Study Protocol - Table of Contents

Study Summary 3
Rationale & background information 4
References (of literature cited in preceding sections) 4
Study aim and objectives 7
Aim 7
Objectives 7
Hypotheses 7
Study Design 7
Overview 7
Methodology 9
Control group – usual care and pamphlet 9
Intervention group – usual care and access to Thought Spot platform 9
Inclusion criteria: 9
Exclusion criteria: 10
Setting 10
Sample size 10
Recruitment 11
Randomization and blinding 12
Data Collection 12
Scales/Measures 12
Digital data collection 13
End-of-study qualitative interviews 13
Withdrawal criteria 13
Safety Considerations and Risks 14
Adequacy of protection against risks 14
Potential Benefits 15
Safety Monitoring 15
Confidentiality 15
REDCap 15
Digital Data 16
Qualitative Interview Data 16
Data Storage 16
Reporting 16
Analysis 16
Power Analysis 16
Statistical Analysis Plan 17
Other Analyses 17
Protocol Change Log 20
Statistical Analysis Plan Change Log 22
References for Change Logs 23

Study Summary

The onset of mental illness across the lifespan is highest among children and youth, with 70% of cases emerging before age 24 [1]. Despite the benefits of early identification and treatment, at-risk members of this age group have great difficulty accessing and receiving the mental health services they need [2-6]. With more than 90% of youth using the Internet [7-9], web-based electronic health (eHealth) and mobile-based health (mHealth) interventions are promising tools for reaching this age group [10-15], potentially bridging the gap for hard-to-reach populations such as young males or other transition-aged youth who have been reluctant to seek services [16-18]. Within this study we define transition-aged youth as those aged 16-29.

Using a partially blinded, two-arm randomized control study design, this project will test the hypothesis that transition-aged youth who use Thought Spot, a youth-driven open-source online and mHealth intervention, will have greater improvement in self-efficacy and intentions towards help-seeking for mental health concerns compared to a control group. The study will also examine whether participants in the intervention arm show greater improvements in health literacy, including awareness of available services and supports, increased self-efficacy in managing their mental health concerns, and a reduction in mental health stigma, compared to the control arm. Participants in the intervention arm will have access to the Thought Spot platform (online and mobile versions), while the control arm will receive usual care (access to campus health services, Web- and print-based information materials).

Our target population for this study is transition aged youth in post-secondary settings. Throughout this document we refer to them as students, participants, and/or youth. To take part in the study, participants must be transition-aged youth [19] of any gender who are currently enrolled in part-time or full-time studies at one of 3 participating postsecondary institutions in the GTA: University of Toronto, Ryerson University, and George Brown College. At baseline, 236 subjects will be recruited for the intervention arm and 236 subjects for the control arm, which amounts to 472 subjects in total. Participants will be compensated for their time. Data will be collected at baseline, 3 and 6 months from all participants through a battery of online questionnaires to measure primary outcomes (changes in help-seeking intentions), help-seeking behaviours and help-seeking attitudes (secondary outcomes) as well as self-reported changes in self-stigma and self-efficacy, data on demographics and general mental health status. The duration of the study is 6 months.

Participants in the intervention arm will additionally complete a usability survey at the 6-month mark to measure usefulness, satisfaction and ease of use of the Thought Spot application. The purpose of the survey is to explore factors related to app acceptance, feasibility and performance that may affect help-seeking intentions, behaviours and attitudes. Based on the usability survey results, a purposeful sample of 15-20 participants who completed this survey will be invited to participate in a 30-minute qualitative interview to better understand enablers and barriers to using Thought Spot and its impact on help-seeking intentions, help-seeking behaviours and attitudes, health literacy, self-efficacy and empowerment. Both study arms will be receiving and completing the exact same set of 6-month survey questionnaires prior to the usability assessments. Therefore, the extra step of usability testing done subsequently with the intervention arm only, should not influence or confound the results.

This study is an innovative randomized control trial, in its testing of a youth-driven mHealth intervention for its effectiveness. The study will extend and advance our understanding of whether mHealth interventions can help bridge the health literacy gap, facilitate increased self-efficacy and lead to appropriate help-seeking for the target population.

Rationale & background information

Seventy per cent of lifetime cases of mental illness will emerge prior to age 24 [1]. While early detection and interventions can address approximately 70% of childhood cases of mental health problems, only about 36% of those youth in need receive mental health support [2]. Within the current social and medical system the act of help-seeking is a relatively unlikely choice for many transition-aged youth [3,4,20,21]. The barriers associated with help-seeking in young adults vary, and are highest when there is lower access to health services, less support from family and friends, and a lower sense of self-worth [22]. The stigma attached to mental illness, as well as embarrassment and fear of confidentiality breaches, are other major barriers associated with help-seeking among young adults [6,10,23-27].

In addition, mental health literacy, a basic understanding of psychological disorders, where to seek mental health information and treatments, and attitudes toward help seeking, is generally poor among transition-aged youth, with many unable to identify the signs of a mental health problem or when professional help is needed [10,28-30]. The occurrence of mental health concerns during this critical developmental period, together with the lack of treatment and support, can result in an array of complications, often including self-medication with alcohol and other drugs, and an inability to thrive socially, academically and vocationally [31].

The gap is clear: 75% of youth are not receiving the services they need [2]. Current avenues for seeking and accessing services are not efficient or effective. With more than 90% of youth using the Internet [7-9], Web-based electronic health (eHealth) and mobile-based health (mHealth) interventions have been identified as promising tools for reaching this age group [10-15], Websites that are easy to navigate and visually engaging are preferred by many young people [32], while interactive elements, such as coaching and crowd-sourcing, are effective and well-received [33]

In collaboration with Ryerson University, OCAD University, University of Toronto (U of T), Faculty of Medicine, ConnexOntario and Kids Help Phone, the Centre for Addiction and Mental Health (CAMH) team worked with over 65 university and college students to develop Thought Spot, a mobile and online resource designed to help increase access to mental health and wellness services. The resource allows users to interact with a crowd-sourced social sharing and navigation platform to find services in their own specific areas using geo-location technology, providing up-to-date information related to services such as primary care, specialized mental health or addictions services, peer support, sexual health, and crisis information, along with wellness-focused services. The goal is to enhance the direct help-seeking intentions, attitudes and behaviours by increasing knowledge of appropriate services for postsecondary students, minimizing the need for intermediaries. Thought Spot makes access to information easy and interactive by mobilizing students to share their knowledge about services, discover wellness options in their area and build peer networks to find mental health and wellness services and supports ('spots') in their vicinity, read reviews and comments from peers, and add their own 'spots'.

References (of literature cited in preceding sections)

1. Kessler RC, Angermeyer M, Anthony JC, Demyttenaere K, Gasquet I, Gluzman S, et al. Lifetime prevalence and age-of-onset distributions of mental disorders in the World Health Organization's World Mental Health Survey Initiative. World Psychiatry 2007 Oct;6(3):168-176 [FREE Full text] [Medline: 18188442]
2. Merikangas KR, He J, Burstein M, Swendsen J, Avenevoli S, Case B, et al. Service utilization for lifetime mental disorders in U.S. adolescents: results of the National Comorbidity Survey-Adolescent Supplement (NCS-A). J Am Acad Child Adolesc Psychiatry 2011 Jan;50(1):32-45 [FREE Full text] [doi: 10.1016/j.jaac.2010.10.006] [Medline: 21156268]

3. Hom MA, Stanley IH, Joiner TE. Evaluating factors and interventions that influence help-seeking and mental health service utilization among suicidal individuals: a review of the literature. Clin Psychol Rev 2015 Aug;40:28-39. [doi:10.1016/j.cpr.2015.05.006] [Medline: 26048165]
4. Michelmore L, Hindley P. Help-seeking for suicidal thoughts and self-harm in young people: a systematic review. Suicide Life Threat Behav 2012 Oct;42(5):507-524. [doi: 10.1111/j.1943-278X.2012.00108.x] [Medline: 22889130]
5. McGorry P, Bates T, Birchwood M. Designing youth mental health services for the 21st century: examples from Australia, Ireland and the UK. Br J Psychiatry Suppl 2013 Jan;54:s30-s35. [doi: 10.1192/bjp.bp.112.119214] [Medline: 23288499]
6. Lee J, Friesen BJ, Walker JS, Colman D, Donlan WE. Youth's help-seeking intentions for ADHD and depression: findings from a national survey. J Child Fam Stud 2012 Dec 12;23(1):144-156. [doi: 10.1007/s10826-012-9700-3]
7. Burns JM, Davenport TA, Durkin LA, Luscombe GM, Hickie IB. The Internet as a setting for mental health service utilization by young people. Med J Aust 2010 Jun 7;192(11 Suppl):S22-S26. [Medline: 20528703]
8. Beck F, Richard JB, Nguyen-Thanh V, Montagni I, Parizot I, Renahy E. Use of the internet as a health information resource among French young adults: results from a nationally representative survey. J Med Internet Res 2014;16(5):e128 [FREE Full text] [doi: 10.2196/jmir.2934] [Medline: 24824164]
9. Lenhart A, Purcell K, Smith A, Zickuhr K. Social media & mobile internet use among teens and young adults. Washington: Pew Research Centre; 2010 Feb 03. URL: http://www.pewinternet.org/files/old-media//Files/Reports/2010/PIP_Social_Media_and_Young_Adults_Report_Final_with_toplines.pdf [accessed 2016-09-01] [WebCite Cache ID 6kCuA81Dg]
10. Czyz EK, Horwitz AG, Eisenberg D, Kramer A, King CA. Self-reported barriers to professional help seeking among college students at elevated risk for suicide. J Am Coll Health 2013;61(7):398-406 [FREE Full text] [doi:10.1080/07448481.2013.820731] [Medline: 24010494]
11. Beck F, Richard JB, Nguyen-Thanh V, Montagni I, Parizot I, Renahy E. Use of the internet as a health information resourceamong French young adults: results from a nationally representative survey. J Med Internet Res 2014;16(5):e128 [FREE Full text] [doi: 10.2196/jmir.2934] [Medline: 24824164]
12. Lal S, Adair CE. E-mental health: a rapid review of the literature. Psychiatr Serv 2014 Jan 1;65(1):24-32. [doi:10.1176/appi.ps.201300009] [Medline: 24081188]
13. Kauer SD, Mangan C, Sanci L. Do online mental health services improve help-seeking for young people? A systematic review. J Med Internet Res 2014;16(3):e66 [FREE Full text] [doi: 10.2196/jmir.3103] [Medline: 24594922]
14. Rice SM, Goodall J, Hetrick SE, Parker AG, Gilbertson T, Amminger GP, et al. Online and social networking interventions for the treatment of depression in young people: a systematic review. J Med Internet Res 2014 Sep 16;16(9):e206 [FREE Full text] [doi: 10.2196/jmir.3304] [Medline: 25226790]
15. Lai MH, Maniam T, Chan LF, Ravindran AV. Caught in the web: a review of Web-based suicide prevention. J Med Internet Res 2014 Jan;16(1):e30 [FREE Full text] [doi: 10.2196/jmir.2973] [Medline: 24472876]
16. Rickwood D, Deane FP, Wilson CJ, Ciarrochi J. Young people's help-seeking for mental health problems. Adv Ment Health 2005 Dec;4(3):218-251. [doi: 10.5172/jamh.4.3.218]
17. Ybarra ML, Eaton WW. Internet-based mental health interventions. Ment Health Serv Res 2005 Jun;7(2):75-87. [Medline:15974154]
18. Ellis LA, Collin P, Hurley PJ, Davenport TA, Burns JM, Hickie IB. Young men's attitudes and behaviour in relation to mental health and technology: implications for the development of online mental health services. BMC Psychiatry 2013 Apr 20;13:119 [FREE Full text] [doi: 10.1186/1471-244X-13-119] [Medline: 23601273]
19. Mental Health Commission of Canada. Taking the Next Step Forward: Building a Responsive Mental Health and Addictions System for Emerging Adults. 2015. URL: http://www.mentalhealthcommission.ca/sites/default/files/Taking%252520the%252520Next%252520Step%252520Forward_0.pdf [accessed 2016-08-30] [WebCite Cache ID 6iTu4OpjP]
20. Gilchrist H, Sullivan G. Barriers to help-seeking in young people: community beliefs about youth suicide. Aust Soc Work 2006 Mar;59(1):73-85. [doi: 10.1080/03124070500449796]

21. Wetterlin FM, Mar MY, Neilson EK, Werker GR, Krausz M. eMental health experiences and expectations: a survey of youths' Web-based resource preferences in Canada. J Med Internet Res 2014;16(12):e293 [FREE Full text] [doi:10.2196/jmir.3526] [Medline: 25519847]
22. Nada-Raja S, Morrison D, Skegg K. A population-based study of help-seeking for self-harm in young adults. Aust N Z J Psychiatry 2003 Oct;37(5):600-605. [Medline: 14511089]
23. Taylor-Rodgers E, Batterham PJ. Evaluation of an online psychoeducation intervention to promote mental health help seeking attitudes and intentions among young adults: randomised controlled trial. J Affect Disord 2014 Oct 15;168:65-71.[doi: 10.1016/j.jad.2014.06.047] [Medline: 25038293]
24. Davidson S, Cappelli M. We've Got Growing Up to Do: Transitioning Youth from Child and Adolescent Mental Health Services to Adult Mental Health Services. Ontario Centre of Excellence for Child and Youth Mental Health. 2011. URL: http://www.excellenceforchildandyouth.ca/sites/default/files/policy_growing_up_to_do.pdf [accessed 2016-04-04] [WebCiteCache ID 6gWXb1T86]
25. Bulanda JJ, Bruhn C, Byro-Johnson T, Zentmyer M. Addressing mental health stigma among young adolescents: evaluation of a youth-led approach. Health Soc Work 2014 May;39(2):73-80. [Medline: 24946423]
26. Brown A, Rice SM, Rickwood DJ, Parker AG. Systematic review of barriers and facilitators to accessing and engaging with mental health care among at-risk young people. Asia Pac Psychiatry 2016 Mar;8(1):3-22. [doi: 10.1111/appy.12199] [Medline: 26238088]
27. Yap MB, Reavley N, Jorm AF. Where would young people seek help for mental disorders and what stops them? Findings from an Australian national survey. J Affect Disord 2013 May;147(1-3):255-261. [doi: 10.1016/j.jad.2012.11.014] [Medline: 23228570]
28. Rickwood D, Deane FP, Wilson CJ, Ciarrochi J. Young people's help-seeking for mental health problems. Adv Ment Health 2005 Dec;4(3):218-251. [doi: 10.5172/jamh.4.3.218]
29. Jorm AF, Korten AE, Jacomb PA, Christensen H, Rodgers B, Pollitt P. “Mental health literacy”: a survey of the public's ability to recognise mental disorders and their beliefs about the effectiveness of treatment. Med J Aust 1997 Feb17;166(4):182-186. [Medline: 9066546]
30. Downs MF, Eisenberg D. Help seeking and treatment use among suicidal college students. J Am Coll Health 2012;60(2):104-114. [doi: 10.1080/07448481.2011.619611] [Medline: 22316407]
31. Hickie I, Groom G, Davenport T. Investing in Australia's Future: The Personal, Social and Economic Benefits of Good Mental Health. Canberra: Mental Health Council of Australia; 2004. URL: https://mhaustralia.org/sites/default/files/imported/component/rsfiles/mental-health-services/Investing_In_Australia_s_Future_Full_Report_.pdf [accessed 2016-08-30] [WebCite Cache ID 6k9QBwrn3]
32. Mental Health Commission of Canada. A Foundation for Online Knowledge Mobilization in Child and Youth Mental Health. Calgary, Canada: Mental Health Commission of Canada; 2012. URL: http://www.mentalhealthcommission.ca/English/system/files/private/document/cy_online_knowledge_mobilization_final_report_eng.pdf [accessed 2016-08-30] [WebCite Cache ID 6gWdbqMdZ]
33. Shandley K, Austin D, Klein B, Kyrios M. An evaluation of 'Reach Out Central': an online gaming program for supporting the mental health of young people. Health Educ Res 2010 Aug;25(4):563-574 [FREE Full text] [doi: 10.1093/her/cyq002] [Medline: 20150530]

Study aim and objectives

Aim

This project will evaluate an mHealth intervention, Thought Spot, designed to enable transition-aged youth in postsecondary settings to identify and overcome barriers to obtaining help for mental health and wellness related issues. Our team will evaluate how Thought Spot enhances participant's help-seeking behaviours for mental health support and services, and thereby increasing their use of appropriate mental health and wellness services. Using a randomized control study design, we will test the hypothesis that transition-aged youth using Thought Spot will have greater improvement in self-efficacy and intentions towards help-seeking for mental health concerns compared to the control group.

Objectives

Primary objective: To evaluate the impact of Thought Spot on intentions in help-seeking for mental health concerns among transition aged youth (youth aged 16-29) enrolled full-time or part-time at a college or university in the Greater Toronto Area (GTA), compared with a control group (who receive usual care; resource pamphlet).

Secondary objective: To examine whether participants in the intervention arm will show greater improvements in health literacy, including awareness of available services and supports, increased self-efficacy in managing their mental health concerns, and a reduction in mental health stigma, compared to the control arm.

Exploratory: To complete an economic evaluation of the Thought Spot intervention compared to usual care to determine the potential cost-effectiveness and financial implications of sustainable and widespread use of Thought Spot throughout Canadian postsecondary campuses.

Hypotheses

(1) Transition-aged youth who receive the intervention will have a greater improvement in intentions and self-efficacy in help-seeking for mental health concerns than those in the control group (usual care; resource pamphlet).

(2) Participants in the intervention arm will also have greater improvements in health literacy, including awareness of available services and supports, increased self-efficacy in managing their mental health concerns, and a reduction in mental health stigma, compared to the control arm.

Study Design

Overview

A two-group partially blinded pre-post randomized controlled study is designed to evaluate the impact of the digital platform, Thought Spot, on transition-aged youths self-efficacy in help-seeking behaviours. To maintain partial blindness, researchers administering surveys will not be aware of the group allocation of participants. A researcher who will not be in contact with participants will conduct the randomization. Measurements will be taken over a 6 month period: baseline, 3 months, and 6 months. 472 participants who are enrolled part-time or full-time at one of 3 participating post-secondary institutions (George Brown College, Ryerson University, University of Toronto) who are interested in maintaining or managing their mental health will be recruited and randomized to the intervention arm or the control arm. Please refer to the study flowchart:

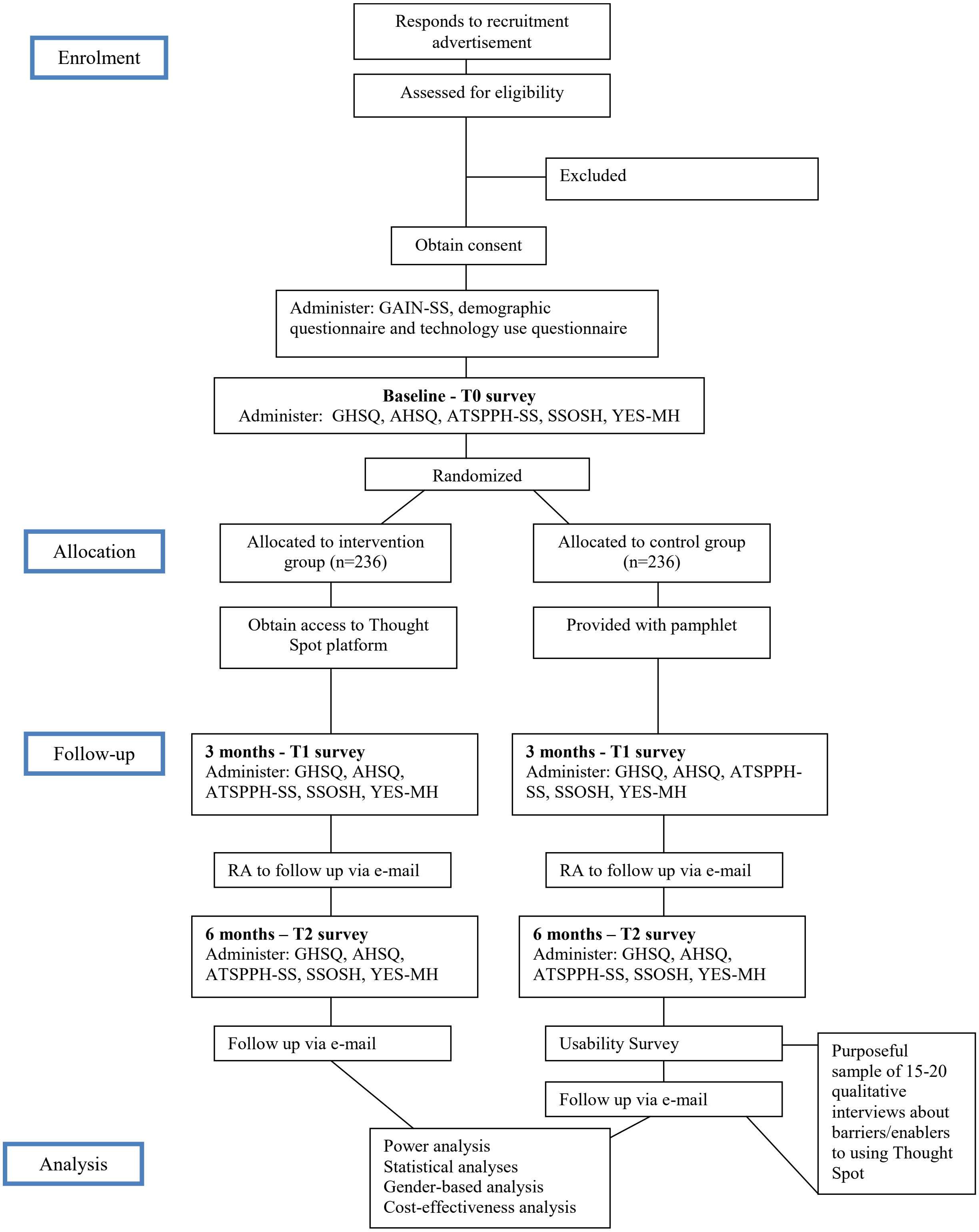

GAIN-SS – Global Appraisal of Individual Needs – Short Screener
GHSQ – General Help Seeking Questionnaire
AHSQ – Actual Help Seeking Questionnaire
ATSPPH-SS – Attitudes towards Seeking Professional Psychological Help – Short Form
SSOSH – Self-Stigma of Seeking Help Scale
YES-MH – Youth Efficacy/Empowerment Scale – Mental Health

Methodology

This is a partially blind study. The process of inviting students and collecting the data will not be done by the researchers who will have contact with students. Missing values will be minimized by reminders sent to participant at least 3 times and the offer of a reward at the end of the 3rd survey. In the invitation stage we will mention the support of the universities, discuss confidentiality, and explain the importance of the research to foster participation and minimize dropouts. We cannot guarantee that control subjects will not interact with intervention subjects causing some contamination, but given the personal nature of mental health conditions we believe this threat will not be highly prevalent. At the analysis stage we will handle missing values with maximum likelihood estimation, which does not disregard dropouts and is unbiased under MAR (Missing at Random) assumption. Demographic variables (e.g., age, gender, academic year) will be included as covariates which should address differences between groups at baseline and improve estimates, even though these differences are not expected due to the randomization.

Control group – usual care and pamphlet

The control group participants will receive a pamphlet that outlines mental health and wellness services across the GTA. Participants within the control group will also have access to usual care including access to campus health services for mental health and wellness and web- and print- based information materials. Customized pamphlets will be created for each participating postsecondary institution. Using equivalent information and headings throughout, the pamphlet will include the main health and wellness program at the designated institution as well as helplines, walk-in services and crisis resources. Since this study revolves around mental health and wellness, our team felt it necessary to provide an additional resource to control group participants. One of the main issues for transition-aged youth is locating and accessing mental health and wellness services. This pamphlet provides information on their school's health and wellness centre as well as general helplines, walk-in counselling services and crisis supports.

Intervention group – usual care and access to Thought Spot platform

The intervention group participants will have access to the Thought Spot platform. This digital platform was designed and produced in partnership with transition-aged youth who are also post-secondary students. The platform maps out wellness and mental health services across the GTA. Users can also privately construct brief journal entries, and connect experiences to moods and locations. All user entries are displayed on a timeline feature within the application that tracks and presents journal entries and services that the user has added to the platform. Private journal entries are not made available to researchers.

Both groups will receive a small compensation for completion of the study (completing all three surveys) to minimize overall attrition.

Inclusion criteria:

1) Aged 17-29 years, inclusive
2) Be enrolled in full-time or part-time studies at one of the participating postsecondary institutions: University of Toronto (St. George and Scarborough), Ryerson University, and George Brown College

3) All participants must have functional competency in English
4) Participants must have a self-reported interest in maintaining or managing their mental health
5) Participants must have access to digital devices compatible with the Thought Spot digital platform.

Inclusion criteria will depend entirely on self-reported information from participants. Research staff will not verify through student records, personal identification, or language tests if participants meet the outlined inclusion criteria.

Exclusion criteria:

1. Individuals who do not have access to a functional mobile device or computer.
2. Individuals who are actively suicidal.

Exclusive criteria will also depend on self-reported information. Once participants consent to the study, they will fill in the Global Appraisal of Individual Needs-Short Screener. This scale collects information on internalizing or externalizing psychiatric disorders, substance use, suicidality, and crime or violence problems. If participants are actively suicidal, they will be immediately referred to crisis support services.

Setting

This RCT will be conducted collaboratively across several partners, and a multi-disciplinary team of experienced health professionals, administrators, researchers and economists, to ensure the successful evaluation of Thought Spot. For the purposes of this project, the primary community will be college and university campuses within the Greater Toronto Area (GTA), including the U of T, Ryerson University and George Brown College.

Sample size

To determine the sample size required to test the primary hypothesis that the Thought Spot intervention will cause a greater change in help seeking intentions compared to usual care, as measured by the difference in formal help seeking intentions from 6 months to baseline, a series of Monte Carlo simulations were carried out (with 10000 replications under each test scenario) using SAS System for Windows v.9.4 (SAS Institute Inc. Cary, NC). These simulations assume that the primary hypothesis will be tested using Mixed Effect Models to account for the longitudinal design and contrasts using time and arm to compare the changes from baseline in both arms. We assume that the test will be two tailed and with critical alpha level of 0.05. In order to simulate the data, means and standard deviation, as well as within subject association are assumed to follow published data that used similar study design. A small effect size, equivalent to a Cohen's d of 0.25, was considered (i.e., an average change in the Help-Seeking Scale of 0.41 - which is a change of 15%), within subject correlation of 0.6, attrition rate of 40% and power of 80%. Based on these simulations and specifications, a sample of 236 subjects per arm before attrition should be used.

To prevent dropping below the 60% response rate required, we will examine our response rates for the final T2 surveys (6 month survey) when the first block of approximately 50-100 participants completes the T2 survey at the 6-month mark of the study. We are proposing to recruit an additional 50 participants if we drop below a 65% response rate at that point. The rationale for this decision is to maintain the power for the analysis in this study.

The participants in the intervention arm will be invited to a usability survey at the 6-month mark which measures usefulness, satisfaction and ease of use of the Thought Spot application. The sample for this survey is limited to the intervention arm because only these participants had access to the Thought Spot application.

A purposeful sample of 15-20 participants who completed the usability survey will be invited to participate in a 30 minute in-person or telephone qualitative interview to better understand enablers and barriers to using Thought Spot and its impact on help-seeking intentions, health literacy, self-efficacy and empowerment.   The purposeful sampling criteria follows:

1) 5-10 participants with lower than average usability study scores and those describing more barriers to use in the open-ended survey questions and/or low-frequency users as indicated by usage reports.

2) 5-10 participants with higher than average usability study scores and those describing more ease with use in the open-ended survey questions and/or, “super-users” as indicated by usage reports.

Given that this is a qualitative study, our aim will be data saturation, that is, the point at which no new information or themes are observed in the data. Typically, saturation can be achieved within 15 semi-structured interviews. Therefore, we propose an initial sample of 10-20 students for the qualitative interviews. If we do not reach saturation once we have reached our participant targets, we will continue to recruit new participants until saturation is achieved.

Recruitment

To recruit 472 participants for the RCT, we will use a number of strategies. The recruitment plan was developed through input from: 1) a Thought Spot student research advisory group (group of 30 students from the thought spot student group); 2) focus groups regarding recruitment for the RCT and 3) individual interviews with campus staff and student leaders. This input was gathered through an earlier stage in the project in which youth were engaged to help co-design the Thought Spot app and the research strategy. An academic literature review was also conducted to identify effective research recruitment strategies for transition-aged youth.

This study will use the following tools: 1) recruitment through invitations/letters circulated through a range campus listservs (e.g.: academic department, health promotion, clubs, student life and course-based listservs); 2) posting of recruitment posters (e.g.: on school bulletin boards, at student centres, student residences, libraries, athletic centres, student common areas, CAMH bulletin boards, LCD screens that have powerpoint slide show played on a loop); 3) placing “app cards” where students pick up print-based information (e.g.: waiting rooms at health and wellness or counselling clinics, student centres, clubs and other common areas); 4) posting the study invitation or advertisement on campus websites (e.g.: health and wellness websites, health promotion websites, student club websites; 5) posting the study invitation or advertisement in student newspapers, student magazines or student blogs; 6) presentations/tables and disseminating swag at campus events (e.g.: health fairs, anti-stigma events, orientation events, club days, etc.); 7) social media promotion (Twitter, Facebook and Instagram); 8) presentations by research team members to student recruiters (e.g.: residence advisors/dons, student mentors, student club leaders/members); 9) psychology research recruitment sites for students; 10) posting the invitation letter through academic courses that offer course credits in exchange for research participation 11) Word of mouth.

Recruitment for the end-of-study qualitative interviews will occur at the 6-month mark of the study. The last question of the end-of-study usability survey will ask if particpants if they would be willing to participate in a 30min in-person or telephone interview about their experiences using Thought Spot (Yes/No) and that additional compensation will be provided. This question will also state that not everyone will be contacted for an interview as spots are limited. Participants will be sampled using a purposeful sampling strategy, described in the Sample Size section, above.

Recruitment supports: The Thought Spot Student Advisory group (TSSG) is a group of transition-aged students who have been participating in the co-creation of the Thought Spot tool throughout the project. They include students in health sciences, design, engineering, health IT. The TSSG will help the research team identify relevant student groups and departments that should be targeted throughout the recruitment process. We will also draw on the expertise of research team members affiliated with the included GTA institutions as well as those institutions'' health promotion teams, to help us gain access to relevant student services, groups and departments (e.g., student residences, health disciplines, health informatics, psychology and public health departments).

Consent process: Once participants have responded to the study advertisements, using a study script, a research analyst assigned to this project will explain the study either in-person or by e-mail outling the study criteria and sending a REDCap link to the consent process. To reduce additional steps during the consent process, participants will be led to a REDCap survey where they will review the inclusion criteria and consent form. Once they've reviewed both components, they will indicate whether they consent to the research study or not. The consent form provides detailed information on the purpose and process of the study, potential risks and confidentiality. If a participant would rather review the consent form in person or by e-mail, they are invited to schedule a meeting with the research analyst.

Randomization and blinding

Through concealed and blocked randomization, the subjects will be randomized to the intervention or control groups. To maintain a partially blinded study, the randomization of participants will be conducted by the research coordinator, who will not conduct data collection or recruitment. Randomization of participants will be set in blocks of 8. Until 4 participants are randomized to one group, the remaining assignments will be to the other group until 8 is complete. This will guarantee a balanced sample size in each study group (intervention and control). Block size will be blinded from researchers conducting recruitment and data collection. This will also eliminate any potential for selection bias and minimize confounding factors. We are using computer-generated random allocation of participants to the intervention and control arms, by means of a randomization module within REDCap. A unique identifier will be assigned to participants after the participant's initial details have been obtained.

Data Collection

Scales/Measures

The following scales will be administered to all participants at baseline, before randomization and separate from other scales: (1) Socio-demographic questionnaire to identify potential confounding factors, during analysis; (2) Global Appraisal of Individual Needs- Short Screener (GAIN-SS) to screen participants as having severe behavioural health disorders, and to measure behavioural health change over study time. The following scales will be administered to all participants at baseline, at the 3 month mark and 6 month mark: (3) General Help Seeking Questionnaire to measure intentions to seek help from different sources and for different problems; (4) Actual Help Seeking Questionnaire to measure past help-seeking experiences; (5) Attitudes Towards Seeking Professional

Psychological Help –Short Form to measure participants process of seeking professional help; (6) Self-Stigma of Seeking Help Scale to measure change in participants self-stigma of seeking psychological help; and (7) Youth Efficacy/Empowerment Scale to measure change in participants empowerment and efficacy when consuming mental health services.

A 6-week reminder and an 18-week reminder will be sent to participants to support their engagement in the study over the 6-month period.

An end-of study usability survey will be administered to participants in the intervention arm at the 6-month mark of the study. This survey measures usefulness, satisfaction and ease of use of the Thought Spot application. The purpose of the survey is to explore factors related to app acceptance, feasibility and performance that may affect help-seeking intentions, behaviours and attitudes of study participants. The survey also asks a short series of demographic questions to identify the respondent's place of residence (downtown Toronto, GTA or other city) because place of residence is a factor in usability. Finally, the survey asks about intentions for future use, whether the respondent would recommend the app to a friend and includes open-ended questions for additional feedback.

Digital data collection
The monitoring of participant data is valuable to the validation of the Thought Spot platform. Digital data will be used to expand on the self-report data collected from participants through the various scales and measures listed above. Usage metrics outlining the intensity of use, usage of content, and methods of accessing app data will be calculated and analyzed. Simple descriptive statistics will also be used to report the time spent on specific app features; number of times the app is used in a day; what times in the day features are being used; and added spots, journal entries, and reviews. This data will be collected from individual, di-identified user accounts. Some of the digital data will also be used to inform app enhancements and plans for development.

End-of-study qualitative interviews
A purposeful sample of 15-20 participants in the intervention group who completed the usability survey will be invited to participate in a 30-60 minute in-person or telephone interview to better understand enablers and barriers to using Thought Spot and its impact on help-seeking intentions, help-seeking behaviours and attitudes, health literacy, self-efficacy and empowerment.

The interviews will be conducted in a semi-structured manner with a set of predetermined open-ended questions and probing questions between interviewer and participant. Interviews will be audio recorded and transcribed. The transcripts will be codified and analyzed for themes related to enablers and barriers to use, app acceptance, feasibility, performance, as well as the impact of Thought Spot on help-seeking intentions, health seeking behaviours and attitudes, health literacy, self-efficacy and empowerment.

Withdrawal criteria
Participants are free to leave the study at any time. If participants decide to stop participating at any point during the study, they may also decide whether they want any of the information that they have

contributed up to that point to be removed or if they will allow us to use that information towards our analysis. Participants will be compensated for the number of completed surveys.

Safety Considerations and Risks

We are aware that some students may also utilize Thought Spot for crisis support. Access to appropriate mental health crisis support will be identified for each university or college recruitment site. A prominent crisis-redirect "help" feature is built into the app for students to use to immediately connect to crisis resources and help lines. Key members of the research team will be able to provide emergent clinical input or direction, should the need arise. These members of the research team will also provide direction and training to ensure appropriate, meaningful, and effective engagement with transition-aged youth.

In addition, students may have concerns or feel vulnerable that personal information, which they enter into the app may be made public through the research process. Students will be provided information through the consent form about what kinds of digital user-information will be gathered from the app for the research.

Finally, students who are in the control arm of the study may feel disappointed or upset that they were not selected to access the app and/or they may have been joining the study due to the need for support in identifying or accessing mental health services or wellness resources. A resource pamphlet will be made available to all students in the control group with information about crisis support services and other mental health and wellness resources across the GTA.

Adequacy of protection against risks

We will be engaging vulnerable individuals in this process, and protocols will be put in place to monitor and respond to participants' needs at various stages in the study. We anticipate that these measures will be adequate to protecting participants for issues that arise.

1) Access to appropriate mental health crisis support will be identified for each university or college recruitment site, and information regarding services will be provided to all participants. Information regarding these crisis support services will be provided to all participants. A prominent crisis-redirect "help" feature is built into the app for students to use to immediately connect them to crisis resource and help lines.

2) Key members of the research team will be able to provide emergent clinical input or direction, should the need arise. These members of the research team will also provide direction and training to ensure appropriate, meaningful, and effective engagement with youth, especially vulnerable youth.

3) Participants will be provided with information about what kinds of digital user-information will be gathered from the app for the research. This data will only consist of aggregated (group-level) data about usage trends, and therefore the data will be anonymous and will not consist of any information tied to specific individuals.

4) A resource pamphlet will be made available to all students in the control group with information about crisis support services and other mental health and wellness resources.

5) Being audio-recorded may feel awkward or embarrassing for some participants. It is disclosed to the participants on the consent form for the end-of-study interviews that their interviews will be audio-recorded. If they are uncomfortable, they can choose not to participate in this aspect of the study. If they start to feel uncomfortable during the interview, we will choose to terminate the interview for the well-being of the participant.

Potential Benefits

There is no guarantee that participants will benefit in any way from taking part in this study. Participants who are in the intervention arm of the study may experience enhanced help-seeking efficacy around mental health and wellness resources and services through the use of this interactive, dynamic, updatable digital engagement platform. Individuals in the control arm may sustain some of the similar gains, simply by signing up for a study about mental health and wellness and being provided a resource pamphlet with listings of mental health and wellness services. This project harnesses the knowledge of the students themselves, to deliver a sustainable approach to activate youth to have productive interactions with health care systems.

The investigators responsible for this study or the CAMH are not conducting this study to receive commercial benefit. However, if this research produces financial returns from a commercialization of the results in the future, participants will not receive any benefit from these returns.

Safety Monitoring

Study investigators will meet monthly to review data confidentiality, adherence to protocol design, recruitment, and participant complaints. They will determine whether there has been any change in the benefit-to-risk ratio of the study. If an adverse event occurs, it will be reported to the research ethics board. If an unexpected adverse event occurs, the investigators will re-assess the risk/benefit ratio of the study; if deemed necessary, they will submit any modifications to all research ethics boards for approval. At the time of REB annual renewal, the PI will submit to the REB the information about the frequency of the monitoring for adverse events and complaints, the dates that meetings took place, a summary of the cumulative adverse events and complaints, external factors or other relevant information that might have an impact on the safety or ethics of the study, final conclusion regarding changes to the anticipated risk/benefit ratio to study participation, and final recommendations related to the continuation, change, or termination of the study.

Confidentiality

REDCap

Data entered in surveys through REDCap, the platform used to house the online surveys can only be accessed with a password known to key research staff working on the project. Only the investigators and key research staff supported by this project will have access to participants’ identities and linked research materials. All data collected through REDCap will be stored in various relational database tables through the use of foreign keys and indexes. This is within a single MySQL database, which is an open source RDBMS (relational database management system). The front end of REDCap is written in PHP, which is widely used, robust, open source scripting language. Setting up the web server and database server and securing the communication of the servers to each other and to the end-user are the responsibilities of the partner institution that is installing REDCap, and thus must be completed prior to installing REDCap. The institution installing REDCap will store all data captured in REDCap on its own

servers. Thus, all project data is stored and hosted there at the local institution, and no project data is ever transmitted at any time by REDCap from that institution to another institution or organization.

To help protect and secure the data stored in REDCap's back end database, the software application employs various methods to protect against malicious users who may attempt to identify and exploit any security vulnerabilities in the system. Such methods will be described here in technical detail. In REDCap, all incoming data gets intentionally filtered, and sanitized. This includes all data submitted in an HTTP Post request and all query string data found in every URL while accessing REDCap, among other modes through which user-defined data gets submitted in the application. Server environment variables that are vulnerable to forgery by users are also checked and sanitized. All user submitted data is properly filtered for any possibly harmful markup tags.

Digital Data

All digital data will be stored on a secured server that is password protected at CAMH and that is part of a system and facility designed to ensure the privacy of this information. A list linking the unique identifier, e-mail login for Thought Spot, with the participant's name and contact information will be stored separately in a password protected file on a password protected CAMH computer that is on a secured server.

Qualitative Interview Data

Confidentiality of all collected data is of primary concern and every effort will be made to ensure data management respects the privacy and confidentiality of participants. The data collected regarding the participants will not be shared with anyone except with the participant's consent or as required by law. Identifiable information such as the participant's name or any other identifying information will be removed from the transcripts. Audio-recordings of interviews will be destroyed after analysis.

Data Storage

All data including paper copies, audio recordings, and transcripts will be securely stored in a locked filing cabinet in a locked office at the Centre for Addiction and Mental Health, 33 Russell Street. All hard copy materials such as participant consent forms will be securely stored in a locked filing cabinet in a locked office at the Centre for Addiction and Mental Health, 33 Russell Street. All data pertaining to a participant's involvement in this study will only be accessible to the research team. The data for this research study will be retained for 25 years. During this time information will be stored in a locked filing cabinet and a password protected computer in a secure room at the Centre for Addiction and Mental Health, 33 Russell Street. After this time, paper information will be shredded and computerized data will be purged and deleted.

Reporting

Participants will not be identified by name in any publication of research results. Results will be published as group data without the use of characteristics that would identify individual participants.

Analysis

Power Analysis

The outcome considered for our power calculation is the average of the GHSQ scale for the formal sources, although similar effect and sample sizes are expected for the informal sources. To determine the sample size required to test the primary hypothesis that the Thought Spot intervention will cause a greater change in help-seeking intentions than usual care, a series of Monte Carlo simulations were

carried out (with 10,000 replications under each test scenario) using SAS System 9.4 for Windows [35]. These simulations assume that the primary hypothesis will be tested using mixed-effect models to account for the longitudinal design, and linear contrasts between time and arm to compare the changes from baseline in both arms. We assume that the test will be 2 tailed and with a critical Cronbach alpha level of 0.05. In order to simulate the data, the means, standard deviations, and within-subject associations are assumed to follow published data that have used similar study designs [36,37]. Based on previous research using the GHSQ [38], a small effect size, equivalent to a Cohen's d of 0.25, was considered (ie, an average change in the GHSQ of 0.41, which is equivalent to a change of 15%), within-subject correlation of 0.6, attrition rate of 40%, and power of 80%. Based on these simulations and specifications, a sample of 236 subjects per arm at baseline is required, which amounts to 472 subjects in total. If 40% attrition is applied to this initial sample, we will be left with 142 subjects per arm (283 in total) after 6 months, at the conclusion of the study.

Statistical Analysis Plan

All analyses will be carried out using SAS System 9.4 for Windows. Statistical tests will be 2 sided, with confidence levels of 0.05. Prior to testing, a series of univariate analyses will be carried out to ensure that model assumptions are met. To address the primary study hypothesis, a mixed-effect model will be used to account for the longitudinal nature of the data, and for attrition. Missing values will be treated with maximum likelihood estimation in SAS PROC MIXED, which uses all available information in the data. Intention-to-treat analysis will be used; all participants will be analyzed as they were originally allocated after randomization. As a sensitivity analysis, the final model will be fitted only with subjects for whom there is complete data. Formal help-seeking score will be the dependent variable, with study groups (intervention and control) and time points as predictors, and relevant socio-demographics collected at baseline as covariates to control for possible confounding.

The interaction between study group and time will be included in the model, and linear contrasts will be used to compare the groups, specifically regarding the change from baseline to the final time point. Similar models will be used to address the exploratory hypotheses, which examine different scales and trends, on the effect of the intervention over time. Bonferroni adjustment will be used to control the Type I error rate if multiple comparisons are desired. Generalized estimating equations will be used for the AHSQ, since this scale is binary.

Other Analyses

A sex- and gender-based analysis will be completed when analyzing data for Phases 1 and 2. An economic evaluation of the Thought Spot intervention compared to usual care will also be explored to determine the potential cost-effectiveness and financial implications of sustainable and widespread use of Thought Spot throughout Canadian postsecondary campuses. The primary outcome to be assessed will be the change in helping-seeking intentions among the target population.

A cost-effectiveness analysis will be conducted to compare the cost and outcome of the intervention arm against usual care. The main output will be an incremental net benefit of the intervention compared to usual care [39] and an incremental cost for one point improvement in the GHSQ in a form of an incremental cost-effectiveness ratio. We will characterize the uncertainty of the findings using 95% confidence interval and a cost-effectiveness acceptability curve [40].

Usefulness, satisfaction, ease of use, acceptance, feasibility and performance will be analyzed through the end-of-study usability survey. Results will be analyzed using means and standard deviations of all respondents for each category of the questionnaire. The proportions of participants reporting high,

medium, or low levels of satisfaction will also be calculated. The open-ended survey questions will be analyzed thematically. The findings may be correlated with the other study surveys and digital data to explore the possible influence of usage, acceptance and perceived functioning of the app upon help-seeking behaviours and help-seeking attitudes of participants.

Qualitative analysis will be conducted for the end-of-study qualitative interviews. All interviews will be audio recorded, transcribed verbatim and uploaded to NVivo. The research team will each read through data for emerging themes to develop a coding scheme. When complete, RA will code transcripts to identify major themes. We will use the thematic analysis process described by Braun & Clarke (41) to review the transcribed interviews, generate codes, develop themes and finally present the findings in a final report. The process is outlined below:

1. Audio-recorded interviews will be transcribed verbatim and uploaded to NVIVO by the RA.
2. The research team will gather to read through several transcripts to identify codes across the whole body of transcripts. These codes identify features of the data that are interesting to the analyst, and refer to a segment of the transcript that refers to the concepts such as enablers and barriers to use, app feasibility, acceptance, performance, help-seeking intentions, help-seeking behaviours and attitudes, health literacy, self-efficacy and empowerment. By the end of this phase the research team will have developed a coding tree in NVIVO .
3. The research team arranges different codes into potential themes, by collating all the relevant coded data extracts within the identified themes.

4. In this step we will conduct two levels of reviews and refinement our themes. Level one involves verifying of all codes within one theme to form a coherent pattern. Level two involves a similar process, but in relation to the entire data set.

5. Detailed analysis of themes to ensure there is no thematic overlap between the themes developed in step four.

6. Producing the final report.

Remaining References:

34. Urbaniak GC, Plous S. Research Randomizer: Random Sampling and Random Assignment Made Easy. 2016. URL: https://www.randomizer.org/ [accessed 2016-07-05] [WebCite Cache ID 6imQMooUl]
35. SAS Institute Inc. SAS: the power to know. 2016. URL: http://www.sas.com/en_ca/home.html [accessed 2016-07-05] [WebCite Cache ID 6imRcWaAo]
36. Smith CL, Shochet IM. The impact of mental health literacy on help-seeking intentions: results of a pilot study with first year psychology students. Int J Ment Health Promot 2011 Jan;13(2):14-20. [doi: 10.1080/14623730.2011.9715652]
37. Costin DL, Mackinnon AJ, Griffiths KM, Batterham PJ, Bennett AJ, Bennett K, et al. Health e-cards as a means of encouraging help seeking for depression among young adults: randomized controlled trial. J Med Internet Res 2009 Oct;11(4):e42 [FREE Full text] [doi: 10.2196/jmir.1294] [Medline: 19850549]
38. Orlowski SK, Lawn S, Venning A, Winsall M, Jones GM, Wyld K, et al. Participatory research as one piece of the puzzle: a systematic review of consumer involvement in design of technology-based youth mental health and well-being interventions. JMIR Hum Factors 2015 Jul 09;2(2):e12 [FREE Full text] [doi: 10.2196/humanfactors.4361] [Medline: 27025279]
39. Hoch, J. S., Briggs, A. H., & Willan, A. R. (2002). Something old, something new, something borrowed, something blue: a framework for the marriage of health econometrics and cost-effectiveness analysis. Health economics, 11(5), 415-430 [doi: 10.1002/hec.678] [Medline: 12112491]

40. Hoch, J. S., Rockx, M. A., & Krahn, A. D. (2006). Using the net benefit regression framework to construct cost-effectiveness acceptability curves: an example using data from a trial of external loop recorders versus Holter monitoring for ambulatory monitoring of. BMC Health Services Research, 6(1), 68. [FREE Full text] [doi: 10.1186/1472-6963-6-68] [Medline: 16756680]
41. Braun, V., Clarke, V. (2006). Using thematic analysis in psychology. Qualitative research in psychology, 3(2), 77-101.

Protocol Change Log

The following amendments were made to the trial protocol after initial approval by the REB.

Protocol Version	Date	Description of Amendments
1.0	25 May 2017	Initial Approval by Research Ethics Board
2.0	3 October 2017	• Randomization/Blinding: Utilization of the randomization module within REDCap instead of a computer-generated random allocation website (Research Randomizer website). These approaches are considered equivalent<br>• Data Collection: Added 6-week and 18-week reminders sent using REDCap online survey platform to support ongoing engagement with study<br>• Safety Monitoring: Removal of monthly review of accrued data as there is no interim analysis in the study
**Participant recruitment for RCT began**		
3.0	8 August 2018	• Study title: Updated title on protocol to official title of the study: "Bridging the gap – Tools for finding health, mental health and wellness resources for university and college students"<br>• Study Summary: An additional usability survey and end-of-study qualitative interviews were added for participants randomized to the intervention arm. As both study arms will still be receiving and completing the same 6-month follow up survey prior to the additional component, it should not influence or confound study results.<br>• Sample Size: Additional verbiage was added to describe and justify the sample size for the usability survey and end-of-study qualitative<br>• Recruitment: Additional verbiage was added to describe the recruitment approaches for the usability survey and end-of-study qualitative interviews. These approaches do not impact the recruitment of the overall RCT.<br>• Scales/Measures: The Usefulness, Satisfaction, and Ease of Use (USE) Questionnaire will be used for assessing usability of Thought Spot and was added to the protocol. The survey will be administered at the end of the study (6-month follow up).<br>• Data collection (End-of-Study Qualitative Interviews): an additional section was added to describe the sampling and data collection approach for the end-of-study qualitative interviews.<br>• Safety Consideration and Risks/Adequacy of protection against risks – Additional information regarding risks and adequacy of protection against risks was added for the end-of-study qualitative interviews

		• Confidentiality (Qualitative interviews, data storage) – Additional information was added to describe the storage and protection of confidentiality for the end-of-study qualitative interviews<br>• Analysis (other analysis) - The analysis approach for the usability surveys (descriptives) and end-of-study qualitative interviews (thematic analysis) was added.
4.0	4 September 2018	• Sample Size – Additional verbiage was added to ensure adequate power is maintained for the analysis of the study. The power for our study was calculated using a 60% response rate. To prevent dropping below the 60% response rate required, we will examine the response rate of our 6-month (T2) follow-up survey in October 2018. If the response rate drops below 65%, we will recruit an additional 50 participants into the study.

Statistical Analysis Plan Change Log

The following clarifications and analysis were made to the Statistical Analysis Plan listed in the preceding protocol and the published protocol paper published (1).

- In addition to the listed primary outcome of formal help-seeking scores as measured by the GHSQ (Page 17), we conducted an analysis of the help-seeking scores from informal sources as measured by the GHSQ as part of secondary analyses.
- Help-seeking behavior, as measured by the AHSQ, is separated into formal and informal sources, and was analyzed separately as part of secondary analyses.
- In the Power Calculation, a minor correction is made. "Cronbach alpha level of 0.05" actually means "Significance Level 0.05"
- Clarification is made to 'Linear Contrast' in the Power Calculation section. The linear contrast mentioned here refers to the difference in change from baseline to the end of the trial. This is the same as our primary test for the efficacy of our outcome
- In our analysis, there was consensus with the research team and literature that gender can affect help-seeking abilities (2, 3). As such, we controlled for gender across all analysis. This may explain part of the variance in the outcome, increasing power. Controlling for gender was not relevant for the conclusions we present as they were the same when not controlling for gender (results not shown).
- To ensure our results are robust, we conducted model diagnostics analysis which includes autoregressive covariance structure instead and in addition to random effect, looking at residuals, Cook's Distance, Leverage and DFBetas statistics to access outliers, atypical and influential points. Several large values were identified and removal of these values had no impact on our conclusion and no meaningful change in model coefficients. We also inspected the data visually for outliers and normality through the liberal use of graphs.
- An extra sensitivity analysis was conducted where baseline variables significantly associated with dropouts at significance level of 0.20 were added to the primary model. Under the direction of our senior statistician, this is considered a better alternative than the one listed in the protocol which uses completers only. That is because mixed effect models adjusted by maximum likelihood can correct biases caused by missing values as long as the missing values are not associated with information not in the model (Missing At Random – MAR – assumption). By adding baseline variables associated with missing values we increase our chances that we are not violating the MAR assumption. The high level 0.20 was chosen so that we minimize the chances of missing important variables, having in mind that in this case we are more concerned with missing important variables than with including unimportant variables. This was only done for the primary outcome, and reported in our paper. The sensitivity analysis described in the protocol, which keeps only completers, was also conducted and the conclusion was the same.
- Multiple comparisons was not conducted so we did not need to use Bonferroni adjustments. Our secondary analyses are conducted with exploratory nature.

- Since there was no evidence for our primary outcome, we did not conduct the sex- and gender-based analysis.
- We conducted two additional exploratory analyses that are important for the completeness of our analysis and may be of interest to researchers.
    - Per-protocol analysis - In the per-protocol data analysis, we repeated our primary analysis holding in the intervention arm only subjects who logged onto Thought Spot more than once were considered, as a definition of compliance. As an extension of this analysis, also looked at the moderation effect of compliance.
    - App Usage Analysis – We conducted a descriptive analysis of usage on Thought Spot.
- Blinding: The senior biostatistician analyzing the data was blinded to the treatment group, except for the per-protocol analysis which was conducted last.

References for Change Logs

1. Wiljer D, Abi-Jaoude A, Johnson A, Ferguson G, Sanches M, Levinson A, et al. Enhancing Self-Efficacy for Help-Seeking Among Transition-Aged Youth in Postsecondary Settings With Mental Health and/or Substance Use Concerns, Using Crowd-Sourced Online and Mobile Technologies: The Thought Spot Protocol. JMIR Res Protoc. 2016;5(4):e201.
2. Liddon L, Kingerlee R, Barry JA. Gender differences in preferences for psychological treatment, coping strategies, and triggers to help-seeking. Br J Clin Psychol. 2018;57(1):42-58.
3. Leong FTL, Zachar P. Gender and opinions about mental illness as predictors of attitudes toward seeking professional psychological help. British Journal of Guidance & Counselling. 2007;27(1):123-32.
