# Supplementary material for: Effects of a Mobile and Web App (Thought Spot) on Mental Health Help-Seeking Among College and University Students: Randomized Controlled Trial
Source: J Med Internet Res. 2020 Oct 30;22(10):e20790. doi: 10.2196/20790 (PMC7665949; doi:10.2196/20790)
Supplement: Multimedia Appendix 2 [file jmir_v22i10e20790_app2.docx]

# Multimedia Appendix 2: Automated Process for Participants Self-Reporting Active Suicidality

After participants gave informed consent, eligible respondents completed a demographics form, technology use questionnaire and the Global Appraisal of Individual Needs Short Screener (GAIN-SS) via REDCap.[1] The GAIN-SS identifies possible mental health and substance use issues, including suicidal ideation, that required further exploration.[1]

If a participant indicated suicidal ideation within the past month, an automated process was triggered: the next set of surveys was temporarily withheld and the participant received an email that offered access to a clinical team member for support and listed crisis numbers. If the participant did not contact research staff within three days, the surveys were re-started and the participant could proceed with the next step of the study. If the participant did contact the research team for support, suspension from the study continued until support was identified and the participant was assessed by research staff or a clinical research team member.

One participant reached out for extra support in response to the automatic email. As per our automated process, a member of our research team responded and provided referrals to clinical support. Consequently, the participant was permitted to continue in the study because they did not meet the exclusion criteria of having active suicidality. The participant chose to continue in the study.

## Reference

1. GAIN Short Screener (GAIN-SS) GAIN Coordinating Centre 2016 [cited 2019 November 15, 2019 ]; Available from: <http://improvingsystems.ca/img/SSA_Implementation_Guide_Jan2017.pdf>.
