# Supplementary material for: Effects of a Mobile and Web App (Thought Spot) on Mental Health Help-Seeking Among College and University Students: Randomized Controlled Trial
Source: J Med Internet Res. 2020 Oct 30;22(10):e20790. doi: 10.2196/20790 (PMC7665949; doi:10.2196/20790)
Supplement: Multimedia Appendix 3 [file jmir_v22i10e20790_app3.docx]

# Multimedia Appendix 3. Formulas to Calculate Effect Sizes for Repeated Measures and Pretest/Posttest Research Designs

The formula below from Morris and Deshon[1] is suggested for repeated measures designs that focus on estimating changes within a person relative to the variability of change scores.

$$d_{RM} = \frac{M_{D, E}}{{SD}_{D, E}}= \frac{{(M}_{post,E}- M_{pre,E})}{{SD}_{D, E}}$$

($d_{RM}$) is the estimated effect size in the experimental group.${(M}_{D, E})$is the sample mean change, ${(M}_{pre, E})$and ${(M}_{post, E})$are the pretest and posttest scores, respectively, for the experimental group.[1] The standard deviation of change scores is represented by (${SD}_{pre, E}$).[1]

Morris and Deshon[1] recommend using the formula below for pretest/posttest designs where homogeneity of pretest variances is assumed. It is described by the sample statistic,

$$d_{IGPP} = \frac{{(M}_{post, E}- M_{pre, E})}{{SD}_{pre, E}}- \frac{{(M}_{post, C}- M_{pre, C})}{{SD}_{pre, E}}$$

Here, ($d_{IGPP}$) is the estimated effect size. ${(M}_{pre, E})$ and ${(M}_{post, E})$are the pretest and posttest scores, respectively, for the experimental group.[1] ${(M}_{pre, C})$ and ${(M}_{post, C})$are the pretest and posttest scores, respectively, for the control group.[1] The standard deviation of change scores is represented by (${SD}_{pre, E}$).[1]

Reference

1. Morris SB, DeShon RP. Combining effect size estimates in meta-analysis with repeated measures and independent-groups designs. Psychol Methods. 2002 Mar;7(1):105-25. PMID: 11928886. doi: 10.1037/1082-989x.7.1.105.
