# Supplementary material for: Effects of a Mobile and Web App (Thought Spot) on Mental Health Help-Seeking Among College and University Students: Randomized Controlled Trial
Source: J Med Internet Res. 2020 Oct 30;22(10):e20790. doi: 10.2196/20790 (PMC7665949; doi:10.2196/20790)
Supplement: Multimedia Appendix 4 [file jmir_v22i10e20790_app4.docx]

# Multimedia Appendix 4. Group-By-Time Interaction for Help-Seeking Behavior (AHSQ) from Informal Sources


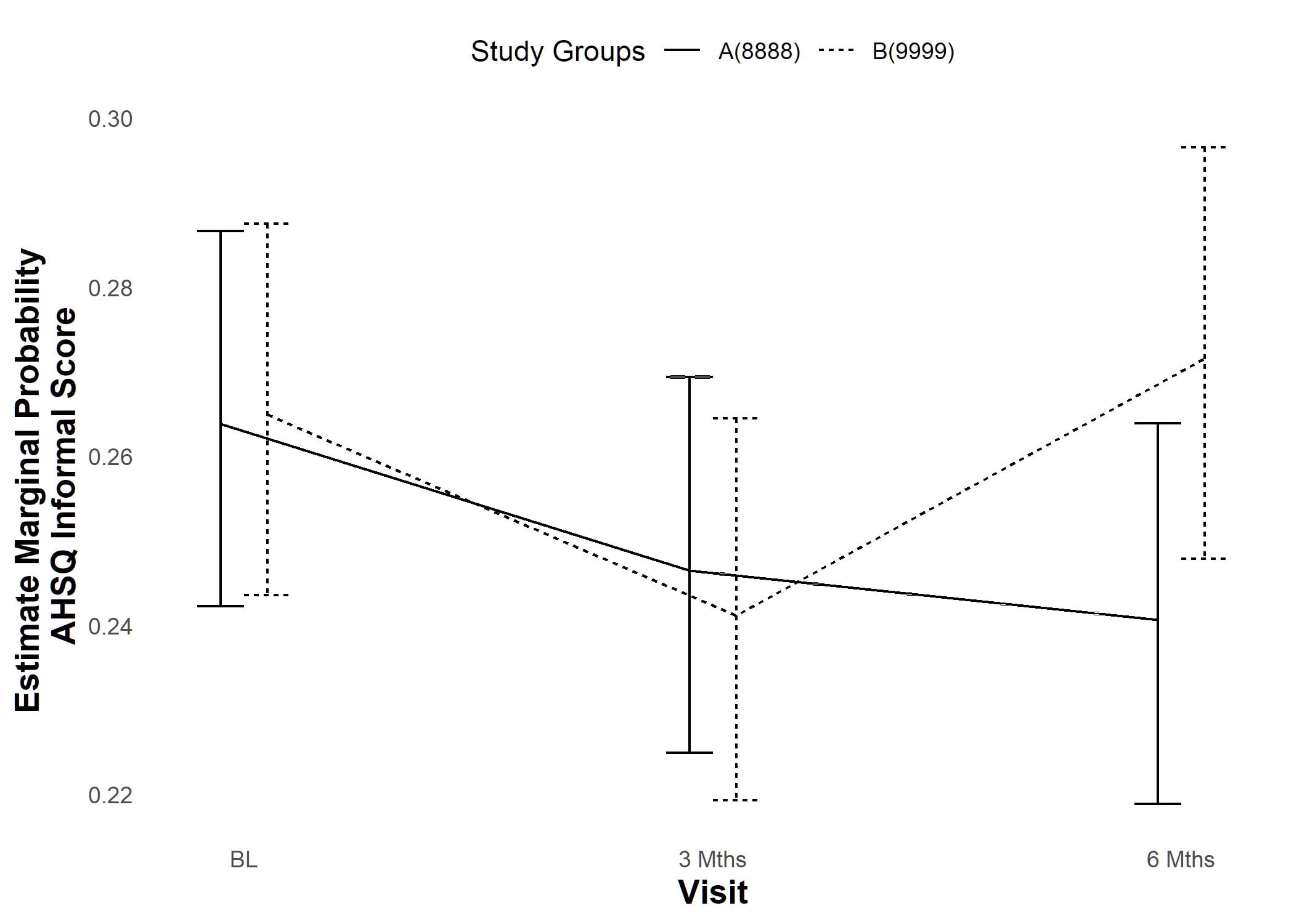


BL denotes the AHSQ score at baseline. Study Group A is the control group while Group B is the intervention group that received Thought Spot.
