# Supplementary material for: Effects of a Mobile and Web App (Thought Spot) on Mental Health Help-Seeking Among College and University Students: Randomized Controlled Trial
Source: J Med Internet Res. 2020 Oct 30;22(10):e20790. doi: 10.2196/20790 (PMC7665949; doi:10.2196/20790)
Supplement: Multimedia Appendix 5 [file jmir_v22i10e20790_app5.docx]

# Multimedia Appendix 5. Gender effects for help-seeking behaviors, intentions and attitudes towards professional help and self-efficacy

| **Survey** | **Gender** | **OR (95% CI)** | **df** | **SE** | **P-Value** |
| --- | --- | --- | --- | --- | --- |
| Formal AHSQ |  |  |  |  |  |
|  | Male (^a^ Ref) | 1.00 |  |  |  |
|  | Female | 1.86 (1.22;2.83) | ^b^ INF | 0.33 | 0.0012* |
|  | Non-Binary | 1.43 (0.55;3.67) | INF | 0.56 | 1.0000 |
| Informal AHSQ |  |  |  |  |  |
|  | Male (Ref) | 1.00 |  |  |  |
|  | Female | 1.29 (1.14;1.46) | INF | 0.07 | <0.001* |
|  | Non-Binary | 1.35 (0.99;1.84) | INF | 0.17 | 0.056 |
| **Survey** | **Gender** | **Estimate (95% CI)** | **df** | **SE** | **P-Value** |
| ATSPPH |  |  |  |  |  |
|  | Male (Ref) | 1.00 |  |  |  |
|  | Female | 0.80 (0.22;1.38) | 475 | 0.24 | 0.003* |
|  | Non-Binary | -1.12 (-2.64;0.40) | 487 | 0.63 | 0.23 |
| SSOSH |  |  |  |  |  |
|  | Male (Ref) | 1.00 |  |  |  |
|  | Female | -0.70 (-1.57;0.18) | 485 | 0.36 | 0.17 |
|  | Non-Binary | 1.25 (-1.05;3.54) | 485 | 0.95 | 0.58 |
| GHSQ Informal |  |  |  |  |  |
|  | Male (Ref) | 1.00 |  |  |  |
|  | Female | -0.14 (-1.26;0.98) | 464 | 0.47 | 1.00 |
|  | Non-Binary | -2.23 (-5.19;0.73) | 462 | 1.23 | 0.21 |
| GHSQ Formal |  |  |  |  |  |
|  | Male (Ref) | 1.00 |  |  |  |
|  | Female | -0.25 (-0.79;0.30) | 484 | 0.23 | 0.83 |
|  | Non-Binary | -1.43 (-2.87; -0.004) | 470 | 0.60 | 0.05 |
|  |  |  |  |  |  |

^a^ Ref denotes the reference used to calculate Odds Ratio

^b^ INF stands for infinity

* Denotes significant p-values that are (p < 0.05)
