# Supplementary material for: Effects of a Mobile and Web App (Thought Spot) on Mental Health Help-Seeking Among College and University Students: Randomized Controlled Trial
Source: J Med Internet Res. 2020 Oct 30;22(10):e20790. doi: 10.2196/20790 (PMC7665949; doi:10.2196/20790)
Supplement: Multimedia Appendix 6 [file jmir_v22i10e20790_app6.docx]

# Multimedia Appendix 6. Gender Effects for help-seeking behaviors, intentions and attitudes towards professional help and self-efficacy when comparing Females and Non-Binary to Males


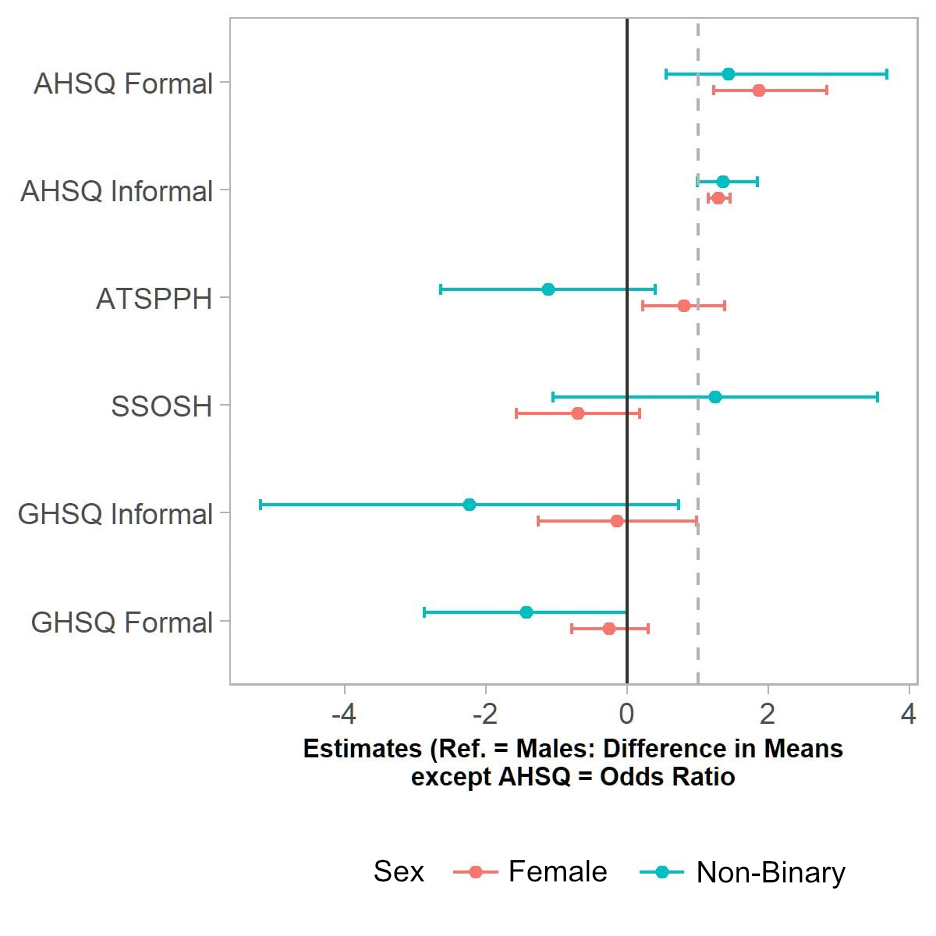


The AHSQ Formal is an odds ratio so the reference is 1, and not 0. Thus, the reference is denoted by the dotted line at 1. All other estimates use 0 as the reference.
