# Supplementary material for: Effects of a Mobile and Web App (Thought Spot) on Mental Health Help-Seeking Among College and University Students: Randomized Controlled Trial
Source: J Med Internet Res. 2020 Oct 30;22(10):e20790. doi: 10.2196/20790 (PMC7665949; doi:10.2196/20790)
Supplement: Multimedia Appendix 7 [file jmir_v22i10e20790_app7.docx]

# Multimedia Appendix 7. Control variables or interactions that were added to the dropout model aside from baseline GHSQ and sex.

| **Data Collection Tool** | **Variable/Interaction** |
| --- | --- |
| Demographics | What devices do you use? Tablet |
|  | What type of smartphone do you have? |
|  | Have you used desktop computer, etc., to: Read or watch the news |
|  | Have you used desktop computer, etc., to: Download an app from a built-in store (Apple App Store, Google Play Store, etc.) |
|  | Financial support: Part-time employment |
|  | Financial support: Parent support |
|  | Financial support: Savings/inheritance |
| ATSPPH | All variables |
| YES | YES Self |
| Global Appraisal of Individual Needs-Short Screener (GAIN-SS) [1] | GAIN Externalizing Disorder Score (EDScr) - 1 Year |
|  | GAIN Substance Use Disorder Score (SDScr) - 1 Year |
|  | GAIN Total Disorders Score (TDScr) - 1 Year |
| Other | Group by Time interaction |

## References

1. GAIN Short Screener (GAIN-SS) GAIN Coordinating Centre 2016 [cited 2019 November 15, 2019 ]; Available from: <http://improvingsystems.ca/img/SSA_Implementation_Guide_Jan2017.pdf>.
