# Supplementary material for: Effects of a Mobile and Web App (Thought Spot) on Mental Health Help-Seeking Among College and University Students: Randomized Controlled Trial
Source: J Med Internet Res. 2020 Oct 30;22(10):e20790. doi: 10.2196/20790 (PMC7665949; doi:10.2196/20790)
Supplement: Multimedia Appendix 8 [file jmir_v22i10e20790_app8.pdf]

CONSORT-EHEALTH (V 1.6.1) - Submission/Publication Form

The CONSORT-EHEALTH checklist is intended for authors of randomized trials evaluating web-based and Internet-based applications/interventions, including mobile interventions, electronic games (incl multiplayer games), social media, certain telehealth applications, and other interactive and/or networked electronic applications. Some of the items (e.g. all subitems under item 5 - description of the intervention) may also be applicable for other study designs.

The goal of the CONSORT EHEALTH checklist and guideline is to be
a) a guide for reporting for authors of RCTs,
b) to form a basis for appraisal of an ehealth trial (in terms of validity)

CONSORT-EHEALTH items/subitems are MANDATORY reporting items for studies published in the Journal of Medical Internet Research and other journals / scientific societies endorsing the checklist.

Items numbered 1., 2., 3., 4a., 4b etc are original CONSORT or CONSORT-NPT (non-pharmacologic treatment) items.
Items with Roman numerals (i., ii, iii, iv etc.) are CONSORT-EHEALTH extensions/clarifications.

As the CONSORT-EHEALTH checklist is still considered in a formative stage, we would ask that you also RATE ON A SCALE OF 1-5 how important/useful you feel each item is FOR THE PURPOSE OF THE CHECKLIST and reporting guideline (optional).

Mandatory reporting items are marked with a red *.
In the textboxes, either copy & paste the relevant sections from your manuscript into this form - please include any quotes from your manuscript in QUOTATION MARKS,
or answer directly by providing additional information not in the manuscript, or elaborating on why the item was not relevant for this study.

YOUR ANSWERS WILL BE PUBLISHED AS A SUPPLEMENTARY FILE TO YOUR PUBLICATION IN JMIR AND ARE CONSIDERED PART OF YOUR PUBLICATION (IF ACCEPTED).
Please fill in these questions diligently. Information will not be copyedited, so please use proper spelling and grammar, use correct capitalization, and avoid abbreviations.

DO NOT FORGET TO SAVE AS PDF _AND_ CLICK THE SUBMIT BUTTON SO YOUR ANSWERS ARE IN OUR DATABASE !!!

Citation Suggestion (if you append the pdf as Appendix we suggest to cite this paper in the caption):
Eysenbach G, CONSORT-EHEALTH Group
CONSORT-EHEALTH: Improving and Standardizing Evaluation Reports of Web-based and

Mobile Health Interventions
J Med Internet Res 2011;13(4):e126
URL: http://www.jmir.org/2011/4/e126/
doi: 10.2196/jmir.1923
PMID: 22209829

* Required

Your name *

First Last

David Wiljer

Primary Affiliation (short), City, Country *

University of Toronto, Toronto, Canada

UHN Digital, Toronto, Canada

Your e-mail address *

abc@gmail.com

david.wiljer@uhn.ca

Title of your manuscript *

Provide the (draft) title of your manuscript.

Effects of a Mobile Application and Online Platform (Thought Spot) on Mental Health Help-Seeking Among College and University Students: A Randomized Controlled Trial

Name of your App/Software/Intervention *

If there is a short and a long/alternate name, write the short name first and add the long name in brackets.

Thought Spot

Evaluated Version (if any)

e.g. "V1", "Release 2017-03-01", "Version 2.0.27913"

Your answer

Language(s) *

What language is the intervention/app in? If multiple languages are available, separate by comma (e.g. "English, French")

English

URL of your Intervention Website or App

e.g. a direct link to the mobile app on app in appstore (itunes, Google Play), or URL of the website. If the intervention is a DVD or hardware, you can also link to an Amazon page.

https://mythoughtspot.ca/

URL of an image/screenshot (optional)

Your answer

Accessibility *

Can an enduser access the intervention presently?

- ○ access is free and open
- ● access only for special usergroups, not open
- ○ access is open to everyone, but requires payment/subscription/in-app purchases
- ○ app/intervention no longer accessible
- ○ Other:

Primary Medical Indication/Disease/Condition *

e.g. "Stress", "Diabetes", or define the target group in brackets after the condition, e.g. "Autism (Parents of children with)", "Alzheimers (Informal Caregivers of)"

Mental Health Help-Seeking

Primary Outcomes measured in trial *

comma-separated list of primary outcomes reported in the trial

Change in formal help-seeking intentions from

Secondary/other outcomes

Are there any other outcomes the intervention is expected to affect?

Changes in informal help-seeking intentions and help-seeking behaviours, help-seeking attitudes, self-stigma and self-efficacy, as measured by the Actual Help-Seeking Questionnaire (AHSQ),the Attitudes Toward Seeking Professional Psychological Help Scale – Short Form (ATSPPH-SF), the Self-Stigma of Seeking Help Scale (SSOSH) and the Youth Efficacy/Empowerment Scale – Mental Health (YES–MH).

Recommended "Dose" *

What do the instructions for users say on how often the app should be used?

- ○ Approximately Daily
- ○ Approximately Weekly
- ○ Approximately Monthly
- ○ Approximately Yearly
- ◉ "as needed"
- ○ Other:

Approx. Percentage of Users (starters) still using the app as recommended after 3 months *

- ○ unknown / not evaluated
- ○ 0-10%
- ◉ 11-20%
- ○ 21-30%
- ○ 31-40%
- ○ 41-50%
- ○ 51-60%
- ○ 61-70%
- ○ 71%-80%
- ○ 81-90%
- ○ 91-100%
- ○ Other:

Overall, was the app/intervention effective? *

- ( ) yes: all primary outcomes were significantly better in intervention group vs control
- ( ) partly: SOME primary outcomes were significantly better in intervention group vs control
- (x) no statistically significant difference between control and intervention
- ( ) potentially harmful: control was significantly better than intervention in one or more outcomes
- ( ) inconclusive: more research is needed
- ( ) Other:

Article Preparation Status/Stage *

At which stage in your article preparation are you currently (at the time you fill in this form)

- ( ) not submitted yet - in early draft status
- (x) not submitted yet - in late draft status, just before submission
- ( ) submitted to a journal but not reviewed yet
- ( ) submitted to a journal and after receiving initial reviewer comments
- ( ) submitted to a journal and accepted, but not published yet
- ( ) published
- ( ) Other:

Journal *

If you already know where you will submit this paper (or if it is already submitted), please provide the journal name (if it is not JMIR, provide the journal name under "other")

- ( ) not submitted yet / unclear where I will submit this
- (•) Journal of Medical Internet Research (JMIR)
- ( ) JMIR mHealth and UHealth
- ( ) JMIR Serious Games
- ( ) JMIR Mental Health
- ( ) JMIR Public Health
- ( ) JMIR Formative Research
- ( ) Other JMIR sister journal
- ( ) Other:

Is this a full powered effectiveness trial or a pilot/feasibility trial? *

- ( ) Pilot/feasibility
- (•) Fully powered

Manuscript tracking number *

If this is a JMIR submission, please provide the manuscript tracking number under "other" (The ms tracking number can be found in the submission acknowledgement email, or when you login as author in JMIR. If the paper is already published in JMIR, then the ms tracking number is the four-digit number at the end of the DOI, to be found at the bottom of each published article in JMIR)

- (•) no ms number (yet) / not (yet) submitted to / published in JMIR
- ( ) Other:

TITLE AND ABSTRACT

1a) TITLE: Identification as a randomized trial in the title

1a) Does your paper address CONSORT item 1a? *

I.e does the title contain the phrase "Randomized Controlled Trial"? (if not, explain the reason under "other")

- (●) yes
- ( ) Other:

1a-i) Identify the mode of delivery in the title

Identify the mode of delivery. Preferably use “web-based” and/or “mobile” and/or “electronic game” in the title. Avoid ambiguous terms like “online”, “virtual”, “interactive”. Use “Internet-based” only if Intervention includes non-web-based Internet components (e.g. email), use “computer-based” or “electronic” only if offline products are used. Use “virtual” only in the context of “virtual reality” (3-D worlds). Use “online” only in the context of “online support groups”. Complement or substitute product names with broader terms for the class of products (such as “mobile” or “smart phone” instead of “iphone”), especially if the application runs on different platforms.

	1	2	3	4	5	
subitem not at all important	( )	( )	(●)	( )	( )	essential

Does your paper address subitem 1a-i? *

Copy and paste relevant sections from manuscript title (include quotes in quotation marks "like this" to indicate direct quotes from your manuscript), or elaborate on this item by providing additional information not in the ms, or briefly explain why the item is not applicable/relevant for your study

Yes, the title is "Effects of a Mobile and Web Application (Thought Spot) on Mental Health Help-Seeking Among College and University Students: A Randomized Controlled Trial"

1a-ii) Non-web-based components or important co-interventions in title

Mention non-web-based components or important co-interventions in title, if any (e.g., “with telephone support”).

	1	2	3	4	5	
subitem not at all important	○	○	○	◉	○	essential

Does your paper address subitem 1a-ii?

Copy and paste relevant sections from manuscript title (include quotes in quotation marks "like this" to indicate direct quotes from your manuscript), or elaborate on this item by providing additional information not in the ms, or briefly explain why the item is not applicable/relevant for your study

This item is not applicable in this study. The app is a standalone intervention.

1a-iii) Primary condition or target group in the title

Mention primary condition or target group in the title, if any (e.g., “for children with Type I Diabetes”)
Example: A Web-based and Mobile Intervention with Telephone Support for Children with Type I Diabetes: Randomized Controlled Trial

	1	2	3	4	5	
subitem not at all important	○	○	○	○	◉	essential

Does your paper address subitem 1a-iii? *

Copy and paste relevant sections from manuscript title (include quotes in quotation marks "like this" to indicate direct quotes from your manuscript), or elaborate on this item by providing additional information not in the ms, or briefly explain why the item is not applicable/relevant for your study

Mental health help-seeking is the primary condition. College and university students is the target population. These are both stated in our manuscript title.

1b) ABSTRACT: Structured summary of trial design, methods, results, and conclusions

NPT extension: Description of experimental treatment, comparator, care providers, centers, and blinding status.

1b-i) Key features/functionalities/components of the intervention and comparator in the METHODS section of the ABSTRACT

Mention key features/functionalities/components of the intervention and comparator in the abstract. If possible, also mention theories and principles used for designing the site. Keep in mind the needs of systematic reviewers and indexers by including important synonyms. (Note: Only report in the abstract what the main paper is reporting. If this information is missing from the main body of text, consider adding it)

	1	2	3	4	5	
subitem not at all important	○	○	◉	○	○	essential

Does your paper address subitem 1b-i? *

Copy and paste relevant sections from the manuscript abstract (include quotes in quotation marks "like this" to indicate direct quotes from your manuscript), or elaborate on this item by providing additional information not in the ms, or briefly explain why the item is not applicable/relevant for your study

Key features of the intervention and comparator are explained in the following way:

"The usual care group received a mental health services information pamphlet. The intervention group received the Thought Spot application on their digital device. Thought Spot is a standalone application that allows users to add, review, and search crowd-sourced information about nearby mental health and wellness services. Users can also track their mood on the app."

1b-ii) Level of human involvement in the METHODS section of the ABSTRACT

Clarify the level of human involvement in the abstract, e.g., use phrases like “fully automated” vs. “therapist/nurse/care provider/physician-assisted” (mention number and expertise of providers involved, if any). (Note: Only report in the abstract what the main paper is reporting. If this information is missing from the main body of text, consider adding it)

	1	2	3	4	5	
subitem not at all important	○	○	○	○	◉	essential

Does your paper address subitem 1b-ii?

Copy and paste relevant sections from the manuscript abstract (include quotes in quotation marks "like this" to indicate direct quotes from your manuscript), or elaborate on this item by providing additional information not in the ms, or briefly explain why the item is not applicable/relevant for your study

Level of human involvement is described like this "Thought Spot is a standalone application that allows users to add, review, and search crowd-sourced information about nearby mental health and wellness services."

1b-iii) Open vs. closed, web-based (self-assessment) vs. face-to-face assessments in the METHODS section of the ABSTRACT

Mention how participants were recruited (online vs. offline), e.g., from an open access website or from a clinic or a closed online user group (closed usergroup trial), and clarify if this was a purely web-based trial, or there were face-to-face components (as part of the intervention or for assessment). Clearly say if outcomes were self-assessed through questionnaires (as common in web-based trials). Note: In traditional offline trials, an open trial (open-label trial) is a type of clinical trial in which both the researchers and participants know which treatment is being administered. To avoid confusion, use “blinded” or “unblinded” to indicated the level of blinding instead of “open”, as “open” in web-based trials usually refers to “open access” (i.e. participants can self-enrol). (Note: Only report in the abstract what the main paper is reporting. If this information is missing from the main body of text, consider adding it)

	1	2	3	4	5	
subitem not at all important	○	○	◉	○	○	essential

Does your paper address subitem 1b-iii?

Copy and paste relevant sections from the manuscript abstract (include quotes in quotation marks "like this" to indicate direct quotes from your manuscript), or elaborate on this item by providing additional information not in the ms, or briefly explain why the item is not applicable/relevant for your study

Outcomes were self-assessed through questionnaires. It is stated in the the abstract like this:

"Outcomes were self-assessed through questionnaires collected at baseline, 3 and 6 months. "

1b-iv) RESULTS section in abstract must contain use data

Report number of participants enrolled/assessed in each group, the use/uptake of the intervention (e.g., attrition/adherence metrics, use over time, number of logins etc.), in addition to primary/secondary outcomes. (Note: Only report in the abstract what the main paper is reporting. If this information is missing from the main body of text, consider adding it)

	1	2	3	4	5	
subitem not at all important	○	○	◉	○	○	essential

Does your paper address subitem 1b-iv?

Copy and paste relevant sections from the manuscript abstract (include quotes in quotation marks "like this" to indicate direct quotes from your manuscript), or elaborate on this item by providing additional information not in the ms, or briefly explain why the item is not applicable/relevant for your study

Use data is mentioned in the results section of the manuscript but not in the abstract like this:

"Of the 241 people randomized to the intervention group, 168 visited Thought Spot, resulting in 3,697 clicks recorded between March 2018 and June 2019. Overall, users viewed 190 “Spots,” conducted 293 searches and created 74 “Thoughts.” “Spots” are locations of mental health and wellness resources. “Thoughts” are users’ personal journal entries on the app. Details of the user data will be published independently of these findings."

1b-v) CONCLUSIONS/DISCUSSION in abstract for negative trials

Conclusions/Discussions in abstract for negative trials: Discuss the primary outcome - if the trial is negative (primary outcome not changed), and the intervention was not used, discuss whether negative results are attributable to lack of uptake and discuss reasons. (Note: Only report in the abstract what the main paper is reporting. If this information is missing from the main body of text, consider adding it)

	1	2	3	4	5	
subitem not at all important	○	○	○	○	◉	essential

Does your paper address subitem 1b-v?

Copy and paste relevant sections from the manuscript abstract (include quotes in quotation marks "like this" to indicate direct quotes from your manuscript), or elaborate on this item by providing additional information not in the ms, or briefly explain why the item is not applicable/relevant for your study

Negative trial is explicitly stated:

"Prompting postsecondary students about mental health and help-seeking appears to increase help-seeking intentions. mHealth interventions may be as effective as information pamphlets in increasing formal help-seeking but may confer a small advantage in driving help-seeking from informal sources. "

INTRODUCTION

2a) In INTRODUCTION: Scientific background and explanation of rationale

2a-i) Problem and the type of system/solution

Describe the problem and the type of system/solution that is object of the study: intended as stand-alone intervention vs. incorporated in broader health care program? Intended for a particular patient population? Goals of the intervention, e.g., being more cost-effective to other interventions, replace or complement other solutions? (Note: Details about the intervention are provided in “Methods” under 5)

	1	2	3	4	5	
subitem not at all important	○	○	○	○	◉	essential

Does your paper address subitem 2a-i? *

Copy and paste relevant sections from the manuscript (include quotes in quotation marks "like this" to indicate direct quotes from your manuscript), or elaborate on this item by providing additional information not in the ms, or briefly explain why the item is not applicable/relevant for your study

The problem is stated like this "Mental health disorders among postsecondary students are a global public health concern.[1-4] Youth aged 16 to 29 years face many challenges in the transition from childhood to adulthood, and 70% of all mental health conditions have their onset during this period.[5-8] Postsecondary education is one experience that can pose many challenges for transition-aged youth due to social, financial and academic stressors.[3, 9-11] Over the last 10 years, rates of mental health disorders among postsecondary students have increased.[12] Yet 35% of youth who are experiencing mental health problems do not seek formal help (e.g., clinical services, health professionals)[13] or informal help (e.g., friends and family, religious leaders, self-help)[13] due to barriers such as perceived stigma, difficulty expressing their concerns, difficulty accessing help and a preference for self-reliance.[14, 15]"

The type of system is explained like this " Applying principles from participatory action and participatory design research,[25, 26] the research team produced Thought Spot, a mHealth intervention co-created with transition-aged youth that aims to improve help-seeking behaviour related to mental health services for postsecondary settings.[27, 28] Thought Spot serves as a map-based database (via a mobile app and website) that allows users to search and geo-locate health, mental health and wellness resources (Figure 1). Additional features of the application include mood and thought tracking, bookmarking searched information, and reading reviews about nearby resources and services. Thought Spot users can also participate in crowdsourcing by adding new resources and writing reviews. "

2a-ii) Scientific background, rationale: What is known about the (type of) system

Scientific background, rationale: What is known about the (type of) system that is the object of the study (be sure to discuss the use of similar systems for other conditions/diagnoses, if appropiate), motivation for the study, i.e. what are the reasons for and what is the context for this specific study, from which stakeholder viewpoint is the study performed, potential impact of findings [2]. Briefly justify the choice of the comparator.

	1	2	3	4	5	
subitem not at all important	○	○	○	◉	○	essential

Does your paper address subitem 2a-ii? *

Copy and paste relevant sections from the manuscript (include quotes in quotation marks "like this" to indicate direct quotes from your manuscript), or elaborate on this item by providing additional information not in the ms, or briefly explain why the item is not applicable/relevant for your study

Scientific background and rationale is explained like this: "Applying principles from participatory action and participatory design research,[25, 26] the research team produced Thought Spot, a mHealth intervention co-created with transition-aged youth that aims to improve help-seeking behaviour related to mental health services for postsecondary settings.[27, 28] Thought Spot serves as a map-based database (via a mobile app and website) that allows users to search and geo-locate health, mental health and wellness resources (Figure 1). Additional features of the application include mood and thought tracking, bookmarking searched information, and reading reviews about nearby resources and services. Thought Spot users can also participate in crowdsourcing by adding new resources and writing reviews."

Mental health information pamphlets were chosen as the comparator because they are the standard source of information about mental health information for students at colleges and universities.

2b) In INTRODUCTION: Specific objectives or hypotheses

Does your paper address CONSORT subitem 2b? *

Copy and paste relevant sections from the manuscript (include quotes in quotation marks "like this" to indicate direct quotes from your manuscript), or elaborate on this item by providing additional information not in the ms, or briefly explain why the item is not applicable/relevant for your study

The objectives and hypotheses are described like this "Accordingly, the main objective of this RCT was to assess the impact of Thought Spot on intentions to seek formal help. The secondary objective was to examine the impact on intentions to seek help from informal sources, and on help-seeking behaviours, help-seeking attitudes, self-stigma and self-efficacy.[17] The research team hypothesized that Thought Spot would be superior to school-specific mental health services information pamphlets in increasing formal help-seeking intentions."

METHODS

3a) Description of trial design (such as parallel, factorial) including allocation ratio

Does your paper address CONSORT subitem 3a? *

Copy and paste relevant sections from the manuscript (include quotes in quotation marks "like this" to indicate direct quotes from your manuscript), or elaborate on this item by providing additional information not in the ms, or briefly explain why the item is not applicable/relevant for your study

Trial Design is described like this: "The team conducted a two-armed, double-blinded RCT"

Allocation Ratio is described like this: "Participants were randomly assigned, using a 1:1 allocation ratio, to either receive access to Thought Spot or to receive a school-specific mental health services information pamphlet. Randomization and allocation was performed using REDCap, a secure, browser-based, electronic data capture system.[29] A randomization module within REDCap provided computer-generated random allocation of participants in blocks of 10."

3b) Important changes to methods after trial commencement (such as eligibility criteria), with reasons

Does your paper address CONSORT subitem 3b? *

Copy and paste relevant sections from the manuscript (include quotes in quotation marks "like this" to indicate direct quotes from your manuscript), or elaborate on this item by providing additional information not in the ms, or briefly explain why the item is not applicable/relevant for your study

There were no important changes to methods after trial commencements to report

3b-i) Bug fixes, Downtimes, Content Changes

Bug fixes, Downtimes, Content Changes: ehealth systems are often dynamic systems. A description of changes to methods therefore also includes important changes made on the intervention or comparator during the trial (e.g., major bug fixes or changes in the functionality or content) (5-iii) and other “unexpected events” that may have influenced study design such as staff changes, system failures/downtimes, etc. [2].

	1	2	3	4	5	
subitem not at all important	○	○	○	○	◉	essential

Does your paper address subitem 3b-i?

Copy and paste relevant sections from the manuscript (include quotes in quotation marks "like this" to indicate direct quotes from your manuscript), or elaborate on this item by providing additional information not in the ms, or briefly explain why the item is not applicable/relevant for your study

Bug fixes, Downtimes, and Content Changes were explained like this "There were no changes to methods or major changes to the features and functionalities of the app. The app content on the app changed by design because participants could add their own crowdsourced information about mental health resources and services during the trial. There were multiple bug fixes to address usability issues related to the malfunctioning buttons, slowness of search features and login credentials not saving. There were no major downtimes during the study period."

4a) Eligibility criteria for participants

Does your paper address CONSORT subitem 4a? *

Copy and paste relevant sections from the manuscript (include quotes in quotation marks "like this" to indicate direct quotes from your manuscript), or elaborate on this item by providing additional information not in the ms, or briefly explain why the item is not applicable/relevant for your study

Eligibility Criteria is described like this: "Full-time and part-time students aged 17–29 years who were enrolled at any of the three postsecondary institutions, who were functionally competent in English and who had access to a digital device compatible with the intervention were eligible to participate in the study. Active suicidality was the sole exclusion criterion, but no participants met this criterion during the screening process"

4a-i) Computer / Internet literacy

Computer / Internet literacy is often an implicit “de facto” eligibility criterion - this should be explicitly clarified.

	1	2	3	4	5	
subitem not at all important	○	○	◉	○	○	essential

Does your paper address subitem 4a-i?

Copy and paste relevant sections from the manuscript (include quotes in quotation marks "like this" to indicate direct quotes from your manuscript), or elaborate on this item by providing additional information not in the ms, or briefly explain why the item is not applicable/relevant for your study

Computer literacy was implied if they had access to a device that was compatible with Thought Spot. This is explained in the manuscript like this

"Full-time and part-time students aged 17–29 years who were enrolled at any of the three postsecondary institutions, who were functionally competent in English and who had access to a digital device compatible with the intervention were eligible to participate in the study. "

4a-ii) Open vs. closed, web-based vs. face-to-face assessments:

Open vs. closed, web-based vs. face-to-face assessments: Mention how participants were recruited (online vs. offline), e.g., from an open access website or from a clinic, and clarify if this was a purely web-based trial, or there were face-to-face components (as part of the intervention or for assessment), i.e., to what degree got the study team to know the participant. In online-only trials, clarify if participants were quasi-anonymous and whether having multiple identities was possible or whether technical or logistical measures (e.g., cookies, email confirmation, phone calls) were used to detect/prevent these.

	1	2	3	4	5	
subitem not at all important	○	○	○	○	◉	essential

Does your paper address subitem 4a-ii? *

Copy and paste relevant sections from the manuscript (include quotes in quotation marks "like this" to indicate direct quotes from your manuscript), or elaborate on this item by providing additional information not in the ms, or briefly explain why the item is not applicable/relevant for your study

The assessment process is explained like this "All participants were enrolled by EH. All authors were blind to the allocation except for JS, who oversaw the randomization process. REDCap was configured to collect data through online questionnaires at baseline, 3 months and 6 months. We asked participants in both groups to use their intervention (Thought Spot or pamphlet) as needed over 6 months. Intervention group participants received detailed instructions via email on how to download and access the Thought Spot app on their personal electronic devices. Participants were also sent two study reminders, at 6 weeks and 18 weeks, in anticipation of the 3-month and 6-month questionnaires. At the end of the study, participants in the intervention group completed an adapted version of the Usefulness, Satisfaction, and Ease of Use (USE) questionnaire,[30] and were invited to participate in qualitative interviews to evaluate Thought Spot's usability and user experience."

4a-iii) Information giving during recruitment

Information given during recruitment. Specify how participants were briefed for recruitment and in the informed consent procedures (e.g., publish the informed consent documentation as appendix, see also item X26), as this information may have an effect on user self-selection, user expectation and may also bias results.

	1	2	3	4	5	
subitem not at all important	◉	○	○	○	○	essential

Does your paper address subitem 4a-iii?

Copy and paste relevant sections from the manuscript (include quotes in quotation marks "like this" to indicate direct quotes from your manuscript), or elaborate on this item by providing additional information not in the ms, or briefly explain why the item is not applicable/relevant for your study

Recruitment procedures are described like this: "Participants were recruited through institutional and student-related listservs, bulletin boards, websites, social media and class presentations." and "Intervention group participants received detailed instructions via email on how to download and access the Thought Spot app on their personal electronic devices. Participants were also sent two study reminders, at 6 weeks and 18 weeks, in anticipation of the 3-month and 6-month questionnaires. "

4b) Settings and locations where the data were collected

Does your paper address CONSORT subitem 4b? *

Copy and paste relevant sections from the manuscript (include quotes in quotation marks "like this" to indicate direct quotes from your manuscript), or elaborate on this item by providing additional information not in the ms, or briefly explain why the item is not applicable/relevant for your study

Setting and locations described like this: The team conducted a two-armed, double-blinded RCT using participants who were students at three postsecondary institutions in the Greater Toronto Area (University of Toronto, Ryerson University, George Brown College)

4b-i) Report if outcomes were (self-)assessed through online questionnaires

Clearly report if outcomes were (self-)assessed through online questionnaires (as common in web-based trials) or otherwise.

	1	2	3	4	5	
subitem not at all important	○	○	○	○	◉	essential

Does your paper address subitem 4b-i? *

Copy and paste relevant sections from the manuscript (include quotes in quotation marks "like this" to indicate direct quotes from your manuscript), or elaborate on this item by providing additional information not in the ms, or briefly explain why the item is not applicable/relevant for your study

Outcomes were self-assessed through online questionnaires. This item is addressed like this in abstract and methods section: "Outcomes were self-assessed through questionnaires collected at baseline, 3 and 6 months."

and

"The primary outcome was a change in formal help-seeking intentions from baseline to 6 months, measured by the General Help Seeking Questionnaire (GHSQ).[31] This scale comprises 10, 7-point Likert scale questions that ask participants how likely they are to seek help from different sources, including friends, family members, mental health professionals and helplines.[31] The formal help-seeking intentions score were calculated by summing the scores of the three questions measuring formal help-seeking intentions.[31] Secondary outcomes of the study included changes in informal help-seeking intentions and help-seeking behaviours, help-seeking attitudes, self-stigma and self-efficacy, measured, respectively, by the Actual Help-Seeking Questionnaire (AHSQ),[31] the Attitudes Toward Seeking Professional Psychological Help Scale – Short Form (ATSPPH-SF),[32] the Self-Stigma of Seeking Help Scale (SSOSH)[33] and the Youth Efficacy/Empowerment Scale – Mental Health (YES–MH).[34]"

4b-ii) Report how institutional affiliations are displayed

Report how institutional affiliations are displayed to potential participants [on ehealth media], as affiliations with prestigious hospitals or universities may affect volunteer rates, use, and reactions with regards to an intervention.(Not a required item – describe only if this may bias results)

	1	2	3	4	5	
subitem not at all important	(•)	( )	( )	( )	( )	essential

Does your paper address subitem 4b-ii?

Copy and paste relevant sections from the manuscript (include quotes in quotation marks "like this" to indicate direct quotes from your manuscript), or elaborate on this item by providing additional information not in the ms, or briefly explain why the item is not applicable/relevant for your study

Your answer

5) The interventions for each group with sufficient details to allow replication, including how and when they were actually administered

5-i) Mention names, credential, affiliations of the developers, sponsors, and owners

Mention names, credential, affiliations of the developers, sponsors, and owners [6] (if authors/evaluators are owners or developer of the software, this needs to be declared in a “Conflict of interest” section or mentioned elsewhere in the manuscript).

	1	2	3	4	5	
subitem not at all important	○	○	○	○	◉	essential

Does your paper address subitem 5-i?

Copy and paste relevant sections from the manuscript (include quotes in quotation marks "like this" to indicate direct quotes from your manuscript), or elaborate on this item by providing additional information not in the ms, or briefly explain why the item is not applicable/relevant for your study

The developers are mentioned in the acknowledgments section like this: "QoC Health served as the software developers of the Thought Spot platform." and the funders of the project are mentioned in the acknowledgments section "QoC Health served as the software developers of the Thought Spot platform."

5-ii) Describe the history/development process

Describe the history/development process of the application and previous formative evaluations (e.g., focus groups, usability testing), as these will have an impact on adoption/use rates and help with interpreting results.

	1	2	3	4	5	
subitem not at all important	○	○	◉	○	○	essential

Does your paper address subitem 5-ii?

Copy and paste relevant sections from the manuscript (include quotes in quotation marks "like this" to indicate direct quotes from your manuscript), or elaborate on this item by providing additional information not in the ms, or briefly explain why the item is not applicable/relevant for your study

History/development process of the application is described like this in the introduction: "Applying principles from participatory action and participatory design research,[25, 26] the research team produced Thought Spot, a mHealth intervention co-created with transition-aged youth that aims to improve help-seeking behaviour related to mental health services for postsecondary settings.[27, 28]"

Additionally, refer to other previously published manuscripts which detail the development process:

1. VanHeerwaarden N, Ferguson G, Abi-Jaoude A, Johnson A, Hollenberg E, Chaim G, et al. The Optimization of an eHealth Solution (Thought Spot) with Transition-Aged Youth in Postsecondary Settings: Participatory Design Research. J Med Internet Res. 2018 Mar 6;20(3):e79. PMID: 29510970. doi: 10.2196/jmir.8102.

2. Wiljer D, Abi-Jaoude A, Johnson A, Ferguson G, Sanches M, Levinson A, et al. Enhancing Self-Efficacy for Help-Seeking Among Transition-Aged Youth in Postsecondary Settings With Mental Health and/or Substance Use Concerns, Using Crowd-Sourced Online and Mobile Technologies: The Thought Spot Protocol. JMIR Res Protoc. 2016 Nov 4;5(4):e201. PMID: 27815232. doi: 10.2196/resprot.6446.

3. Sennah S, Shi J, Hollenberg E, Johnson A, Ferguson G, Abi-Jaoude A, et al. Thought Spot: Embedding Usability Testing into the Development Cycle. Stud Health Technol Inform. 2019;257:375-81. PMID: 30741226.

5-iii) Revisions and updating

Revisions and updating. Clearly mention the date and/or version number of the application/intervention (and comparator, if applicable) evaluated, or describe whether the intervention underwent major changes during the evaluation process, or whether the development and/or content was “frozen” during the trial. Describe dynamic components such as news feeds or changing content which may have an impact on the replicability of the intervention (for unexpected events see item 3b).

	1	2	3	4	5	
subitem not at all important	○	◉	○	○	○	essential

Does your paper address subitem 5-iii?

Copy and paste relevant sections from the manuscript (include quotes in quotation marks "like this" to indicate direct quotes from your manuscript), or elaborate on this item by providing additional information not in the ms, or briefly explain why the item is not applicable/relevant for your study

Your answer

5-iv) Quality assurance methods

Provide information on quality assurance methods to ensure accuracy and quality of information provided [1], if applicable.

	1	2	3	4	5	
subitem not at all important	○	○	○	◉	○	essential

Does your paper address subitem 5-iv?

Copy and paste relevant sections from the manuscript (include quotes in quotation marks "like this" to indicate direct quotes from your manuscript), or elaborate on this item by providing additional information not in the ms, or briefly explain why the item is not applicable/relevant for your study

Quality assurance was conducted through three ways. The research team did our best to validate the content of the app prior to the RCT, which was performed by JS. Additionally, the research team held workshops with students to curate and validate mental health and wellness resources that were included in Thought Spot. The workshops are detailed in a previously published article:

VanHeerwaarden N, Ferguson G, Abi-Jaoude A, Johnson A, Hollenberg E, Chaim G, et al. The Optimization of an eHealth Solution (Thought Spot) with Transition-Aged Youth in Postsecondary Settings: Participatory Design Research. J Med Internet Res. 2018 Mar 6;20(3):e79. PMID: 29510970. doi: 10.2196/jmir.8102.

Also, the crowdsourcing feature allowed users to review and provide feedback on the validity of newly added information during the trial.

5-v) Ensure replicability by publishing the source code, and/or providing screenshots/screen-capture video, and/or providing flowcharts of the algorithms used

Ensure replicability by publishing the source code, and/or providing screenshots/screen-capture video, and/or providing flowcharts of the algorithms used. Replicability (i.e., other researchers should in principle be able to replicate the study) is a hallmark of scientific reporting.

	1	2	3	4	5	
subitem not at all important	◉	○	○	○	○	essential

Does your paper address subitem 5-v?

Copy and paste relevant sections from the manuscript (include quotes in quotation marks "like this" to indicate direct quotes from your manuscript), or elaborate on this item by providing additional information not in the ms, or briefly explain why the item is not applicable/relevant for your study

Your answer

5-vi) Digital preservation

Digital preservation: Provide the URL of the application, but as the intervention is likely to change or disappear over the course of the years; also make sure the intervention is archived (Internet Archive, webcitation.org, and/or publishing the source code or screenshots/videos alongside the article). As pages behind login screens cannot be archived, consider creating demo pages which are accessible without login.

	1	2	3	4	5	
subitem not at all important	○	◉	○	○	○	essential

Does your paper address subitem 5-vi?

Copy and paste relevant sections from the manuscript (include quotes in quotation marks "like this" to indicate direct quotes from your manuscript), or elaborate on this item by providing additional information not in the ms, or briefly explain why the item is not applicable/relevant for your study

Your answer

5-vii) Access

Access: Describe how participants accessed the application, in what setting/context, if they had to pay (or were paid) or not, whether they had to be a member of specific group. If known, describe how participants obtained “access to the platform and Internet” [1]. To ensure access for editors/reviewers/readers, consider to provide a “backdoor” login account or demo mode for reviewers/readers to explore the application (also important for archiving purposes, see vi).

	1	2	3	4	5	
subitem not at all important	○	○	○	◉	○	essential

Does your paper address subitem 5-vii? *

Copy and paste relevant sections from the manuscript (include quotes in quotation marks "like this" to indicate direct quotes from your manuscript), or elaborate on this item by providing additional information not in the ms, or briefly explain why the item is not applicable/relevant for your study

Participants were only given access to the application if they were assigned to the intervention group. Each participant in the intervention group was given unique login information and instructions on how to download and use the application.

5-viii) Mode of delivery, features/functionalities/components of the intervention and comparator, and the theoretical framework

Describe mode of delivery, features/functionalities/components of the intervention and comparator, and the theoretical framework [6] used to design them (instructional strategy [1], behaviour change techniques, persuasive features, etc., see e.g., [7, 8] for terminology). This includes an in-depth description of the content (including where it is coming from and who developed it) [1],” whether [and how] it is tailored to individual circumstances and allows users to track their progress and receive feedback” [6]. This also includes a description of communication delivery channels and – if computer-mediated communication is a component – whether communication was synchronous or asynchronous [6]. It also includes information on presentation strategies [1], including page design principles, average amount of text on pages, presence of hyperlinks to other resources, etc. [1].

	1	2	3	4	5	
subitem not at all important	○	○	○	○	◉	essential

Does your paper address subitem 5-viii? *

Copy and paste relevant sections from the manuscript (include quotes in quotation marks "like this" to indicate direct quotes from your manuscript), or elaborate on this item by providing additional information not in the ms, or briefly explain why the item is not applicable/relevant for your study

The mode of delivery/features/functionalities/components of the intervention are described in the following way:

"Applying principles from participatory action and participatory design research,[25, 26] the research team produced Thought Spot, a mHealth intervention co-created with transition-aged youth that aims to improve help-seeking behaviour related to mental health services for postsecondary settings.[27, 28] Thought Spot serves as a map-based database (via a mobile app and website) that allows users to search and geo-locate health, mental health and wellness resources (Figure 1). Additional features of the application include mood and thought tracking, bookmarking searched information, and reading reviews about nearby resources and services. Thought Spot users can also participate in crowdsourcing by adding new resources and writing reviews. "

5-ix) Describe use parameters

Describe use parameters (e.g., intended "doses" and optimal timing for use). Clarify what instructions or recommendations were given to the user, e.g., regarding timing, frequency, heaviness of use, if any, or was the intervention used ad libitum.

	1	2	3	4	5	
subitem not at all important	○	○	○	●	○	essential

Does your paper address subitem 5-ix?

Copy and paste relevant sections from the manuscript (include quotes in quotation marks "like this" to indicate direct quotes from your manuscript), or elaborate on this item by providing additional information not in the ms, or briefly explain why the item is not applicable/relevant for your study

The use parameters are described like this in the manuscript "We asked participants in both groups to use their intervention (Thought Spot or pamphlet) as needed over 6 months. Intervention group participants received detailed instructions via email on how to download and access the Thought Spot app on their personal electronic devices.

5-x) Clarify the level of human involvement

Clarify the level of human involvement (care providers or health professionals, also technical assistance) in the e-intervention or as co-intervention (detail number and expertise of professionals involved, if any, as well as “type of assistance offered, the timing and frequency of the support, how it is initiated, and the medium by which the assistance is delivered”. It may be necessary to distinguish between the level of human involvement required for the trial, and the level of human involvement required for a routine application outside of a RCT setting (discuss under item 21 – generalizability).

	1	2	3	4	5	
subitem not at all important	○	○	○	◉	○	essential

Does your paper address subitem 5-x?

Copy and paste relevant sections from the manuscript (include quotes in quotation marks "like this" to indicate direct quotes from your manuscript), or elaborate on this item by providing additional information not in the ms, or briefly explain why the item is not applicable/relevant for your study

The intervention was intended to be a standalone app and not a co-intervention with care providers or health professionals. Thus, the involvement of care providers and health professionals was minimal.

5-xi) Report any prompts/reminders used

Report any prompts/reminders used: Clarify if there were prompts (letters, emails, phone calls, SMS) to use the application, what triggered them, frequency etc. It may be necessary to distinguish between the level of prompts/reminders required for the trial, and the level of prompts/reminders for a routine application outside of a RCT setting (discuss under item 21 – generalizability).

	1	2	3	4	5	
subitem not at all important	○	○	○	◉	○	essential

Does your paper address subitem 5-xi? *

Copy and paste relevant sections from the manuscript (include quotes in quotation marks "like this" to indicate direct quotes from your manuscript), or elaborate on this item by providing additional information not in the ms, or briefly explain why the item is not applicable/relevant for your study

There were two study reminders sent to participants, which are described in the manuscript like this:

"Participants were also sent two study reminders, at 6 weeks and 18 weeks, in anticipation of the 3-month and 6-month questionnaires."

5-xii) Describe any co-interventions (incl. training/support)

Describe any co-interventions (incl. training/support): Clearly state any interventions that are provided in addition to the targeted eHealth intervention, as ehealth intervention may not be designed as stand-alone intervention. This includes training sessions and support [1]. It may be necessary to distinguish between the level of training required for the trial, and the level of training for a routine application outside of a RCT setting (discuss under item 21 – generalizability.

	1	2	3	4	5	
subitem not at all important	○	○	○	◉	○	essential

Does your paper address subitem 5-xii? *

Copy and paste relevant sections from the manuscript (include quotes in quotation marks "like this" to indicate direct quotes from your manuscript), or elaborate on this item by providing additional information not in the ms, or briefly explain why the item is not applicable/relevant for your study

There were no co-interventions that were provided in addition to the targeted eHealth intervention.

6a) Completely defined pre-specified primary and secondary outcome measures, including how and when they were assessed

Does your paper address CONSORT subitem 6a? *

Copy and paste relevant sections from the manuscript (include quotes in quotation marks "like this" to indicate direct quotes from your manuscript), or elaborate on this item by providing additional information not in the ms, or briefly explain why the item is not applicable/relevant for your study

The primary and secondary outcomes are described in the manuscript like this:

"The primary outcome was a change in formal help-seeking intentions from baseline to 6 months, measured by the General Help Seeking Questionnaire (GHSQ).[31] This scale comprises 10, 7-point Likert scale questions that ask participants how likely they are to seek help from different sources, including friends, family members, mental health professionals and helplines.[31] The formal help-seeking intentions score were calculated by summing the scores of the three questions measuring formal help-seeking intentions.[31] Secondary outcomes of the study included changes in informal help-seeking intentions and help-seeking behaviours, help-seeking attitudes, self-stigma and self-efficacy, measured, respectively, by the Actual Help-Seeking Questionnaire (AHSQ),[31] the Attitudes Toward Seeking Professional Psychological Help Scale – Short Form (ATSPPH-SF),[32] the Self-Stigma of Seeking Help Scale (SSOSH)[33] and the Youth Efficacy/Empowerment Scale – Mental Health (YES–MH).[34]"

6a-i) Online questionnaires: describe if they were validated for online use and apply CHERRIES items to describe how the questionnaires were designed/deployed

If outcomes were obtained through online questionnaires, describe if they were validated for online use and apply CHERRIES items to describe how the questionnaires were designed/deployed [9].

	1	2	3	4	5	
subitem not at all important	○	○	◉	○	○	essential

Does your paper address subitem 6a-i?

Copy and paste relevant sections from manuscript text

Your answer

6a-ii) Describe whether and how “use” (including intensity of use/dosage) was defined/measured/monitored

Describe whether and how “use” (including intensity of use/dosage) was defined/measured/monitored (logins, logfile analysis, etc.). Use/adoption metrics are important process outcomes that should be reported in any ehealth trial.

	1	2	3	4	5	
subitem not at all important	○	○	○	◉	○	essential

Does your paper address subitem 6a-ii?

Copy and paste relevant sections from manuscript text

This item is important and usage data was monitored and analyzed. A high-level overview of usage data is stated in the manuscript like this

"Of the 241 people randomized to the intervention group, 168 visited Thought Spot, resulting in 3,697 clicks recorded between March 2018 and June 2019. Overall, users viewed 190 “Spots,” conducted 293 searches and created 74 “Thoughts.” “Spots” are locations of mental health and wellness resources. “Thoughts” are users’ personal journal entries on the app. Details of the user data will be published independently of these findings."

An in-depth analysis was also performed on the usage data, but the detailed findings of this analysis will be reported in a separate manuscript.

6a-iii) Describe whether, how, and when qualitative feedback from participants was obtained

Describe whether, how, and when qualitative feedback from participants was obtained (e.g., through emails, feedback forms, interviews, focus groups).

	1	2	3	4	5	
subitem not at all important	○	○	○	◉	○	essential

Does your paper address subitem 6a-iii?

Copy and paste relevant sections from manuscript text

Qualitative feedback was collected in the following way:

"At the end of the study, participants in the intervention group completed an adapted version of the Usefulness, Satisfaction, and Ease of Use (USE) questionnaire,[30] and were invited to participate in qualitative interviews to evaluate Thought Spot’s usability and user experience."

6b) Any changes to trial outcomes after the trial commenced, with reasons

Does your paper address CONSORT subitem 6b? *

Copy and paste relevant sections from the manuscript (include quotes in quotation marks "like this" to indicate direct quotes from your manuscript), or elaborate on this item by providing additional information not in the ms, or briefly explain why the item is not applicable/relevant for your study

There were no changes to trial outcomes after the trial commenced

7a) How sample size was determined

NPT: When applicable, details of whether and how the clustering by care provides or centers was addressed

7a-i) Describe whether and how expected attrition was taken into account when calculating the sample size

Describe whether and how expected attrition was taken into account when calculating the sample size.

	1	2	3	4	5	
subitem not at all important	○	○	○	○	◉	essential

Does your paper address subitem 7a-i?

Copy and paste relevant sections from manuscript title (include quotes in quotation marks "like this" to indicate direct quotes from your manuscript), or elaborate on this item by providing additional information not in the ms, or briefly explain why the item is not applicable/relevant for your study

Sample size was calculated in the following way:

"To determine the required sample size, power calculations were conducted using the primary outcome – the average formal help-seeking score on the GHSQ.[27] Monte Carlo simulations were conducted in SAS 9.4 (Windows) using the means, standard deviation and within-subject associations reported in previous research using the GHSQ.[35, 36] The calculation accounted for a 40% dropout rate and assumed the use of a mixed-effects model to test the primary hypothesis at a significance level of 0.05 (two-tailed). A sample of 472 participants (236 per arm) was found to provide 80% power to detect a small effect size (Cohen’s d=0.25), which is equivalent to a 15% difference in change in GHSQ score from baseline to 6 months."

7b) When applicable, explanation of any interim analyses and stopping guidelines

Does your paper address CONSORT subitem 7b? *

Copy and paste relevant sections from the manuscript (include quotes in quotation marks "like this" to indicate direct quotes from your manuscript), or elaborate on this item by providing additional information not in the ms, or briefly explain why the item is not applicable/relevant for your study

Not Applicable

8a) Method used to generate the random allocation sequence

NPT: When applicable, how care providers were allocated to each trial group

Does your paper address CONSORT subitem 8a? *

Copy and paste relevant sections from the manuscript (include quotes in quotation marks "like this" to indicate direct quotes from your manuscript), or elaborate on this item by providing additional information not in the ms, or briefly explain why the item is not applicable/relevant for your study

The randomization method is described in the following way:

"Randomization and allocation was performed using REDCap, a secure, browser-based, electronic data capture system.[29] A randomization module within REDCap provided computer-generated random allocation of participants in blocks of 10."

8b) Type of randomisation; details of any restriction (such as blocking and block size)

Does your paper address CONSORT subitem 8b? *

Copy and paste relevant sections from the manuscript (include quotes in quotation marks "like this" to indicate direct quotes from your manuscript), or elaborate on this item by providing additional information not in the ms, or briefly explain why the item is not applicable/relevant for your study

The type of randomization and block size is described like this:

"Randomization and allocation was performed using REDCap, a secure, browser-based, electronic data capture system.[29] A randomization module within REDCap provided computer-generated random allocation of participants in blocks of 10"

9) Mechanism used to implement the random allocation sequence (such as sequentially numbered containers), describing any steps taken to conceal the sequence until interventions were assigned

Does your paper address CONSORT subitem 9? *

Copy and paste relevant sections from the manuscript (include quotes in quotation marks "like this" to indicate direct quotes from your manuscript), or elaborate on this item by providing additional information not in the ms, or briefly explain why the item is not applicable/relevant for your study

Mechanism used to implement random allocation sequence is described below:

Randomization and allocation was performed using REDCap, a secure, browser-based, electronic data capture system.[29] A randomization module within REDCap provided computer-generated random allocation of participants in blocks of 10. All participants were enrolled by EH. All authors were blind to the allocation except for JS, who oversaw the randomization process.

10) Who generated the random allocation sequence, who enrolled participants, and who assigned participants to interventions

Does your paper address CONSORT subitem 10? *

Copy and paste relevant sections from the manuscript (include quotes in quotation marks "like this" to indicate direct quotes from your manuscript), or elaborate on this item by providing additional information not in the ms, or briefly explain why the item is not applicable/relevant for your study

This CONSORT sub-item is described in the manuscript like this:

"A randomization module within REDCap provided computer-generated random allocation of participants in blocks of 10. All participants were enrolled by EH. All authors were blind to the allocation except for JS, who oversaw the randomization process. "

11a) If done, who was blinded after assignment to interventions (for example, participants, care providers, those assessing outcomes) and how

NPT: Whether or not administering co-interventions were blinded to group assignment

11a-i) Specify who was blinded, and who wasn’t

Specify who was blinded, and who wasn’t. Usually, in web-based trials it is not possible to blind the participants [1, 3] (this should be clearly acknowledged), but it may be possible to blind outcome assessors, those doing data analysis or those administering co-interventions (if any).

	1	2	3	4	5	
subitem not at all important	○	○	○	○	◉	essential

Does your paper address subitem 11a-i? *

Copy and paste relevant sections from the manuscript (include quotes in quotation marks "like this" to indicate direct quotes from your manuscript), or elaborate on this item by providing additional information not in the ms, or briefly explain why the item is not applicable/relevant for your study

Blinded individuals are described in the following ways:

"All authors were blind to the allocation except for JS, who oversaw the randomization process."

and

"Statistical analysis was performed by a biostatistician (MS) who was blinded to randomization."

11a-ii) Discuss e.g., whether participants knew which intervention was the “intervention of interest” and which one was the “comparator”

Informed consent procedures (4a-ii) can create biases and certain expectations - discuss e.g., whether participants knew which intervention was the “intervention of interest” and which one was the “comparator”.

	1	2	3	4	5	
subitem not at all important	○	◉	○	○	○	essential

Does your paper address subitem 11a-ii?

Copy and paste relevant sections from the manuscript (include quotes in quotation marks "like this" to indicate direct quotes from your manuscript), or elaborate on this item by providing additional information not in the ms, or briefly explain why the item is not applicable/relevant for your study

This item is addressed like this: ". Group assignment could not be blinded for participants."

11b) If relevant, description of the similarity of interventions

(this item is usually not relevant for ehealth trials as it refers to similarity of a placebo or sham intervention to a active medication/intervention)

Does your paper address CONSORT subitem 11b? *

Copy and paste relevant sections from the manuscript (include quotes in quotation marks "like this" to indicate direct quotes from your manuscript), or elaborate on this item by providing additional information not in the ms, or briefly explain why the item is not applicable/relevant for your study

N/A

12a) Statistical methods used to compare groups for primary and secondary outcomes

NPT: When applicable, details of whether and how the clustering by care providers or centers was addressed

Does your paper address CONSORT subitem 12a? *

Copy and paste relevant sections from the manuscript (include quotes in quotation marks "like this" to indicate direct quotes from your manuscript), or elaborate on this item by providing additional information not in the ms, or briefly explain why the item is not applicable/relevant for your study

Primary and Secondary outcomes were analyzed using the following statistical methods detailed in the manuscript:

"Univariate analyses were conducted to describe groups at baseline (Table 1) and to compare completers and non-completers of the questionnaires. Completers were defined as participants who finished the survey at all three points of the study.[27] To evaluate the primary hypothesis, the research team used mixed-effects models, using the intention-to-treat method, where time, group and gender were entered as fixed effects and participants were entered as random effects. A linear contrast of estimated marginal means from the fixed-effect interaction between time and group was conducted to compare changes from baseline to 6 months across groups. Missing values were handled by maximum likelihood estimation, which is able to incorporate all available information in the data, under the missing at random assumptions.[37] The team conducted sensitivity analyses, in which we added model variables found to have significant bivariate associations with dropouts, defined as participants who were randomized but who did not complete all 3 surveys, at a significance level of α=0.20. "

"Similar models were conducted to explore the secondary outcomes of this study, except for help-seeking behaviour. This outcome was measured using the binary-scaled AHSQ and was analysed using a mixed binomial logistic regression model. The p-values for the secondary analysis were not adjusted for multiple comparison. "

12a-i) Imputation techniques to deal with attrition / missing values

Imputation techniques to deal with attrition / missing values: Not all participants will use the intervention/comparator as intended and attrition is typically high in ehealth trials. Specify how participants who did not use the application or dropped out from the trial were treated in the statistical analysis (a complete case analysis is strongly discouraged, and simple imputation techniques such as LOCF may also be problematic [4]).

	1	2	3	4	5	
subitem not at all important	○	○	○	●	○	essential

Does your paper address subitem 12a-i? *

Copy and paste relevant sections from the manuscript (include quotes in quotation marks "like this" to indicate direct quotes from your manuscript), or elaborate on this item by providing additional information not in the ms, or briefly explain why the item is not applicable/relevant for your study

Missing values procedures were handled using the following method: "Missing values were handled by maximum likelihood estimation, which is able to incorporate all available information in the data, under the missing at random assumptions.[37]"

12b) Methods for additional analyses, such as subgroup analyses and adjusted analyses

Does your paper address CONSORT subitem 12b? *

Copy and paste relevant sections from the manuscript (include quotes in quotation marks "like this" to indicate direct quotes from your manuscript), or elaborate on this item by providing additional information not in the ms, or briefly explain why the item is not applicable/relevant for your study

Methods for secondary analyses and per-protocol analyses are stated like this in the manuscript:

"In addition to the prespecified analysis plan, the research team conducted an exploratory per-protocol analysis of the primary and secondary outcomes. Participants who logged onto Thought Spot more than once for the duration of the study were considered compliant."

Further details are described in Multimedia Appendix 1 - Protocol and Statistical Analysis Plan

X26) REB/IRB Approval and Ethical Considerations [recommended as subheading under "Methods"] (not a CONSORT item)

X26-i) Comment on ethics committee approval

	1	2	3	4	5	
subitem not at all important	○	○	○	○	◉	essential

Does your paper address subitem X26-i?

Copy and paste relevant sections from the manuscript (include quotes in quotation marks "like this" to indicate direct quotes from your manuscript), or elaborate on this item by providing additional information not in the ms, or briefly explain why the item is not applicable/relevant for your study

REB Approval is described in the methods section like this: "The protocol was approved by the research ethics boards of each participating postsecondary institution and the Centre for Addiction and Mental Health and is detailed in Supplement 1. Digital informed consent was obtained from each participant through REDCap before participants could be included in the trial.[29]"

x26-ii) Outline informed consent procedures

Outline informed consent procedures e.g., if consent was obtained offline or online (how? Checkbox, etc.?), and what information was provided (see 4a-ii). See [6] for some items to be included in informed consent documents.

	1	2	3	4	5	
subitem not at all important	○	○	○	○	◉	essential

Does your paper address subitem X26-ii?

Copy and paste relevant sections from the manuscript (include quotes in quotation marks "like this" to indicate direct quotes from your manuscript), or elaborate on this item by providing additional information not in the ms, or briefly explain why the item is not applicable/relevant for your study

Informed consent procedures are described like this: "Digital informed consent was obtained from each participant through REDCap before participants could be included in the trial.[29]"

X26-iii) Safety and security procedures

Safety and security procedures, incl. privacy considerations, and any steps taken to reduce the likelihood or detection of harm (e.g., education and training, availability of a hotline)

	1	2	3	4	5	
subitem not at all important	○	○	○	○	◉	essential

Does your paper address subitem X26-iii?

Copy and paste relevant sections from the manuscript (include quotes in quotation marks "like this" to indicate direct quotes from your manuscript), or elaborate on this item by providing additional information not in the ms, or briefly explain why the item is not applicable/relevant for your study

Active suicidality was the sole exclusion criterion, and the safety and security procedures for this exclusion criteria is described in Multi-media Appendix 2 like this:

"If a participant indicated active suicidality within the past month, an automated process was triggered: the next set of questions was temporarily withheld and the participant received an email that offered access to a clinical team member for support and listed crisis numbers. If the participant did not contact research staff within three days, the surveys were re-started and the participant could proceed with the next step of the study. If the participant did contact the research team for support, suspension from the study continued until the participant was assessed by a clinical research team member. "

RESULTS

13a) For each group, the numbers of participants who were randomly assigned, received intended treatment, and were analysed for the primary outcome

NPT: The number of care providers or centers performing the intervention in each group and the number of patients treated by each care provider in each center

Does your paper address CONSORT subitem 13a? *

Copy and paste relevant sections from the manuscript (include quotes in quotation marks "like this" to indicate direct quotes from your manuscript), or elaborate on this item by providing additional information not in the ms, or briefly explain why the item is not applicable/relevant for your study

The number of participants who were randomly assigned, received intended treatment, and were analyzed for the primary outcome are listed in Table 1. Baseline Characteristics of Participants Who Underwent Randomization

13b) For each group, losses and exclusions after randomisation, together with reasons

Does your paper address CONSORT subitem 13b? (NOTE: Preferably, this is shown in a CONSORT flow diagram) *

Copy and paste relevant sections from the manuscript (include quotes in quotation marks "like this" to indicate direct quotes from your manuscript), or elaborate on this item by providing additional information not in the ms, or briefly explain why the item is not applicable/relevant for your study

Figure 2 of the manuscript is the CONSORT flow diagram.

13b-i) Attrition diagram

Strongly recommended: An attrition diagram (e.g., proportion of participants still logging in or using the intervention/comparator in each group plotted over time, similar to a survival curve) or other figures or tables demonstrating usage/dose/engagement.

	1	2	3	4	5	
subitem not at all important	○	○	○	◉	○	essential

Does your paper address subitem 13b-i?

Copy and paste relevant sections from the manuscript or cite the figure number if applicable (include quotes in quotation marks "like this" to indicate direct quotes from your manuscript), or elaborate on this item by providing additional information not in the ms, or briefly explain why the item is not applicable/relevant for your study

The attrition information is important. However, we will be reporting this information separately. In this paper Of the 241 people randomized to the intervention group, 168 visited Thought Spot, resulting in 3,697 clicks recorded between March 2018 and June 2019. Overall, users viewed 190 “Spots,” conducted 293 searches and created 74 “Thoughts.” “Spots” are locations of mental health and wellness resources. “Thoughts” are users’ personal journal entries on the app. Details of the user data will be published independently of these findings.

14a) Dates defining the periods of recruitment and follow-up

Does your paper address CONSORT subitem 14a? *

Copy and paste relevant sections from the manuscript (include quotes in quotation marks "like this" to indicate direct quotes from your manuscript), or elaborate on this item by providing additional information not in the ms, or briefly explain why the item is not applicable/relevant for your study

The recruitment process is described like this: "From March 2018 to January 2019, 481 were randomized participants from three Canadian postsecondary institutions into a 6-month trial on a rolling basis. Of these participants, 240 were assigned to the control group and 241 were assigned to the intervention group (Figure 2). The trial ended after all participants received their 6-month follow-up on June 30, 2019. "

14a-i) Indicate if critical “secular events” fell into the study period

Indicate if critical “secular events” fell into the study period, e.g., significant changes in Internet resources available or “changes in computer hardware or Internet delivery resources”

	1	2	3	4	5	
subitem not at all important	○	◉	○	○	○	essential

Does your paper address subitem 14a-i?

Copy and paste relevant sections from the manuscript (include quotes in quotation marks "like this" to indicate direct quotes from your manuscript), or elaborate on this item by providing additional information not in the ms, or briefly explain why the item is not applicable/relevant for your study

Your answer

14b) Why the trial ended or was stopped (early)

Does your paper address CONSORT subitem 14b? *

Copy and paste relevant sections from the manuscript (include quotes in quotation marks "like this" to indicate direct quotes from your manuscript), or elaborate on this item by providing additional information not in the ms, or briefly explain why the item is not applicable/relevant for your study

This consort item is addressed like this: "). The trial ended after all participants received their 6-month follow-up on June 30, 2019. "

15) A table showing baseline demographic and clinical characteristics for each group

NPT: When applicable, a description of care providers (case volume, qualification, expertise, etc.) and centers (volume) in each group

Does your paper address CONSORT subitem 15? *

Copy and paste relevant sections from the manuscript (include quotes in quotation marks "like this" to indicate direct quotes from your manuscript), or elaborate on this item by providing additional information not in the ms, or briefly explain why the item is not applicable/relevant for your study

Demographics are reported in Table 1 of the manuscript.

15-i) Report demographics associated with digital divide issues

In ehealth trials it is particularly important to report demographics associated with digital divide issues, such as age, education, gender, social-economic status, computer/Internet/ehealth literacy of the participants, if known.

	1	2	3	4	5	
subitem not at all important	○	○	○	○	◉	essential

Does your paper address subitem 15-i? *

Copy and paste relevant sections from the manuscript (include quotes in quotation marks "like this" to indicate direct quotes from your manuscript), or elaborate on this item by providing additional information not in the ms, or briefly explain why the item is not applicable/relevant for your study

Age, gender, education, and experience with mental health or substance use concerns are reported in the demographics Table 1.

16) For each group, number of participants (denominator) included in each analysis and whether the analysis was by original assigned groups

16-i) Report multiple “denominators” and provide definitions

Report multiple “denominators” and provide definitions: Report N’s (and effect sizes) “across a range of study participation [and use] thresholds” [1], e.g., N exposed, N consented, N used more than x times, N used more than y weeks, N participants “used” the intervention/comparator at specific pre-defined time points of interest (in absolute and relative numbers per group). Always clearly define “use” of the intervention.

	1	2	3	4	5	
subitem not at all important	○	○	○	◉	○	essential

Does your paper address subitem 16-i? *

Copy and paste relevant sections from the manuscript (include quotes in quotation marks "like this" to indicate direct quotes from your manuscript), or elaborate on this item by providing additional information not in the ms, or briefly explain why the item is not applicable/relevant for your study

The definition of "use" is stated in the manuscript like this:

"Participants who logged onto Thought Spot more than once for the duration of the study were considered compliant. "

The number of participants (denominator) included in each analysis are detailed in results and in Table 2: Main Results from Mixed Models. It is described like this: "240 were assigned to the control group and 241 were assigned to the intervention group".

For the per-protocol analysis. the number of participants (denominator) included in the analysis is stated like this

"Of the 241 participants in the intervention group, 169 (70.1%) met the definition of compliance."

16-ii) Primary analysis should be intent-to-treat

Primary analysis should be intent-to-treat, secondary analyses could include comparing only "users", with the appropriate caveats that this is no longer a randomized sample (see 18-i).

	1	2	3	4	5	
subitem not at all important	○	○	○	○	◉	essential

Does your paper address subitem 16-ii?

Copy and paste relevant sections from the manuscript (include quotes in quotation marks "like this" to indicate direct quotes from your manuscript), or elaborate on this item by providing additional information not in the ms, or briefly explain why the item is not applicable/relevant for your study

Primary analysis is based on intent-to-treat, as stated in Multimedia Appendix 1, page 17 like this: "Intention-to-treat analysis will be used; all participants will be analyzed as they were originally allocated after randomization"

17a) For each primary and secondary outcome, results for each group, and the estimated effect size and its precision (such as 95% confidence interval)

Does your paper address CONSORT subitem 17a? *

Copy and paste relevant sections from the manuscript (include quotes in quotation marks "like this" to indicate direct quotes from your manuscript), or elaborate on this item by providing additional information not in the ms, or briefly explain why the item is not applicable/relevant for your study

Results of each primary and secondary outcome, the estimated effect size and it's precision is included in Table 2 Main Results from Mixed Models (AHSQ)

17a-i) Presentation of process outcomes such as metrics of use and intensity of use

In addition to primary/secondary (clinical) outcomes, the presentation of process outcomes such as metrics of use and intensity of use (dose, exposure) and their operational definitions is critical. This does not only refer to metrics of attrition (13-b) (often a binary variable), but also to more continuous exposure metrics such as “average session length”. These must be accompanied by a technical description how a metric like a “session” is defined (e.g., timeout after idle time) [1] (report under item 6a).

	1	2	3	4	5	
subitem not at all important	○	○	○	◉	○	essential

Does your paper address subitem 17a-i?

Copy and paste relevant sections from the manuscript (include quotes in quotation marks "like this" to indicate direct quotes from your manuscript), or elaborate on this item by providing additional information not in the ms, or briefly explain why the item is not applicable/relevant for your study

This item is important and usage data was monitored and analyzed. A high-level overview of usage data is stated in the manuscript like this

"Of the 241 people randomized to the intervention group, 168 visited Thought Spot, resulting in 3,697 clicks recorded between March 2018 and June 2019. Overall, users viewed 190 “Spots,” conducted 293 searches and created 74 “Thoughts.” “Spots” are locations of mental health and wellness resources. “Thoughts” are users’ personal journal entries on the app. Details of the user data will be published independently of these findings."

An in-depth analysis was also performed on the usage data, but the detailed findings of this analysis will be reported in a separate manuscript.

17b) For binary outcomes, presentation of both absolute and relative effect sizes is recommended

Does your paper address CONSORT subitem 17b? *

Copy and paste relevant sections from the manuscript (include quotes in quotation marks "like this" to indicate direct quotes from your manuscript), or elaborate on this item by providing additional information not in the ms, or briefly explain why the item is not applicable/relevant for your study

Yes, absolute and relative effect sizes are reported as both absolute and relative effect sizes, like in Table 2 Main Results from Mixed Models (AHSQ)

18) Results of any other analyses performed, including subgroup analyses and adjusted analyses, distinguishing pre-specified from exploratory

Does your paper address CONSORT subitem 18? *

Copy and paste relevant sections from the manuscript (include quotes in quotation marks "like this" to indicate direct quotes from your manuscript), or elaborate on this item by providing additional information not in the ms, or briefly explain why the item is not applicable/relevant for your study

Secondary Analyses and per-protocol analyses are reported. Per-protocol analyses were exploratory, as stated in the methods section "In addition to the prespecified analysis plan, the research team conducted an exploratory per-protocol analysis of the primary and secondary outcomes."

18-i) Subgroup analysis of comparing only users

A subgroup analysis of comparing only users is not uncommon in ehealth trials, but if done, it must be stressed that this is a self-selected sample and no longer an unbiased sample from a randomized trial (see 16-iii).

	1	2	3	4	5	
subitem not at all important	○	○	○	●	○	essential

Does your paper address subitem 18-i?

Copy and paste relevant sections from the manuscript (include quotes in quotation marks "like this" to indicate direct quotes from your manuscript), or elaborate on this item by providing additional information not in the ms, or briefly explain why the item is not applicable/relevant for your study

We conducted a per-protocol analysis

19) All important harms or unintended effects in each group

(for specific guidance see CONSORT for harms)

Does your paper address CONSORT subitem 19? *

Copy and paste relevant sections from the manuscript (include quotes in quotation marks "like this" to indicate direct quotes from your manuscript), or elaborate on this item by providing additional information not in the ms, or briefly explain why the item is not applicable/relevant for your study

Harms and unintended effects are described like this in the manuscript: "There were no reported adverse events or harms during the trial."

19-i) Include privacy breaches, technical problems

Include privacy breaches, technical problems. This does not only include physical "harm" to participants, but also incidents such as perceived or real privacy breaches [1], technical problems, and other unexpected/unintended incidents. "Unintended effects" also includes unintended positive effects [2].

	1	2	3	4	5	
subitem not at all important	○	○	○	●	○	essential

Does your paper address subitem 19-i?

Copy and paste relevant sections from the manuscript (include quotes in quotation marks "like this" to indicate direct quotes from your manuscript), or elaborate on this item by providing additional information not in the ms, or briefly explain why the item is not applicable/relevant for your study

The technical problems encountered by participants are described like this:

"There were multiple bug fixes to address usability issues, such as malfunctioning buttons, slowness of search features and login credentials not saving. There were no major downtimes during the study period."

There were no privacy breaches or privacy-related issues that occurred during the study

19-ii) Include qualitative feedback from participants or observations from staff/researchers

Include qualitative feedback from participants or observations from staff/researchers, if available, on strengths and shortcomings of the application, especially if they point to unintended/unexpected effects or uses. This includes (if available) reasons for why people did or did not use the application as intended by the developers.

	1	2	3	4	5	
subitem not at all important	○	○	○	◉	○	essential

Does your paper address subitem 19-ii?

Copy and paste relevant sections from the manuscript (include quotes in quotation marks "like this" to indicate direct quotes from your manuscript), or elaborate on this item by providing additional information not in the ms, or briefly explain why the item is not applicable/relevant for your study

Qualitative feedback from the study participants were analyzed separately and will be reported separately.

DISCUSSION

22) Interpretation consistent with results, balancing benefits and harms, and considering other relevant evidence

NPT: In addition, take into account the choice of the comparator, lack of or partial blinding, and unequal expertise of care providers or centers in each group

22-i) Restate study questions and summarize the answers suggested by the data, starting with primary outcomes and process outcomes (use)

Restate study questions and summarize the answers suggested by the data, starting with primary outcomes and process outcomes (use).

	1	2	3	4	5	
subitem not at all important	○	○	○	◉	○	essential

Does your paper address subitem 22-i? *

Copy and paste relevant sections from the manuscript (include quotes in quotation marks "like this" to indicate direct quotes from your manuscript), or elaborate on this item by providing additional information not in the ms, or briefly explain why the item is not applicable/relevant for your study

Study question and summary of answers suggested by the data is described like this:

" In the analysis of our primary outcome, there were no significant differences in the formal help-seeking intentions of postsecondary students between control and intervention groups. However, both groups experienced a similar increase in formal help-seeking intentions during the 6-month study period, as assessed by the GHSQ. "

and

"Analyses of secondary outcomes revealed no significant group-by-time interactions for help-seeking intentions from informal sources, attitudes toward seeking professional help, self-efficacy, or self-stigma. However, a small increase in help-seeking behaviour related to informal sources between 3 and 6 months was observed with the intervention group, whereas a small decrease was seen in the control group.

22-ii) Highlight unanswered new questions, suggest future research

Highlight unanswered new questions, suggest future research.

	1	2	3	4	5	
subitem not at all important	○	○	○	◉	○	essential

Does your paper address subitem 22-ii?

Copy and paste relevant sections from the manuscript (include quotes in quotation marks "like this" to indicate direct quotes from your manuscript), or elaborate on this item by providing additional information not in the ms, or briefly explain why the item is not applicable/relevant for your study

Future research is suggested in the conclusions: "It is increasingly important as a next step to compare the cost-effectiveness of Thought Spot and information pamphlets for understanding the feasibility and sustainability of mHealth tools compared with existing strategies"

20) Trial limitations, addressing sources of potential bias, imprecision, and, if relevant, multiplicity of analyses

20-i) Typical limitations in ehealth trials

Typical limitations in ehealth trials: Participants in ehealth trials are rarely blinded. Ehealth trials often look at a multiplicity of outcomes, increasing risk for a Type I error. Discuss biases due to non-use of the intervention/usability issues, biases through informed consent procedures, unexpected events.

	1	2	3	4	5	
subitem not at all important	○	○	○	◉	○	essential

Does your paper address subitem 20-i? *

Copy and paste relevant sections from the manuscript (include quotes in quotation marks "like this" to indicate direct quotes from your manuscript), or elaborate on this item by providing additional information not in the ms, or briefly explain why the item is not applicable/relevant for your study

The study limitations are described like this: "The study had some limitations. Group assignment could not be blinded for participants. There were also software bugs that led to an inconsistent app environment and to usability issues during the trial. These issues could have affected the level of user engagement and compliance with the intervention, and ultimately the effectiveness of Thought Spot because some participants had difficulty accessing key functions of the app during certain points of the trial. Additionally, the effect size for the change in informal help-seeking behaviour was small and was noted only in the 3- to 6-month period. Further investigation and a longer follow-up period are required to evaluate the sustained impact of mHealth solutions on informal help-seeking. Finally, the development of Thought Spot and the findings from this trial are based on the unique experiences of transition-aged youth enrolled in Canadian postsecondary institutions and may not be generalizable to youth outside Canadian academic settings."

21) Generalisability (external validity, applicability) of the trial findings

NPT: External validity of the trial findings according to the intervention, comparators, patients, and care providers or centers involved in the trial

21-i) Generalizability to other populations

Generalizability to other populations: In particular, discuss generalizability to a general Internet population, outside of a RCT setting, and general patient population, including applicability of the study results for other organizations

	1	2	3	4	5	
subitem not at all important	○	○	◉	○	○	essential

Does your paper address subitem 21-i?

Copy and paste relevant sections from the manuscript (include quotes in quotation marks "like this" to indicate direct quotes from your manuscript), or elaborate on this item by providing additional information not in the ms, or briefly explain why the item is not applicable/relevant for your study

Limits to the generalizability of this study is stated in the manuscript like this:

"Finally, the development of Thought Spot and the findings from this trial are based on the unique experiences of transition-aged youth enrolled in Canadian postsecondary institutions and may not be generalizable to youth outside Canadian academic settings."

21-ii) Discuss if there were elements in the RCT that would be different in a routine application setting

Discuss if there were elements in the RCT that would be different in a routine application setting (e.g., prompts/reminders, more human involvement, training sessions or other co-interventions) and what impact the omission of these elements could have on use, adoption, or outcomes if the intervention is applied outside of a RCT setting.

	1	2	3	4	5	
subitem not at all important	○	○	◉	○	○	essential

Does your paper address subitem 21-ii?

Copy and paste relevant sections from the manuscript (include quotes in quotation marks "like this" to indicate direct quotes from your manuscript), or elaborate on this item by providing additional information not in the ms, or briefly explain why the item is not applicable/relevant for your study

The key difference between the study and routine application setting would be access. During the study, the participants were given login credentials access by the research team after they consent and randomization procedures. However, in routine settings, a user will be able to freely download the application through the App Store or Google Play store without engaging our research team.

OTHER INFORMATION

23) Registration number and name of trial registry

Does your paper address CONSORT subitem 23? *

Copy and paste relevant sections from the manuscript (include quotes in quotation marks "like this" to indicate direct quotes from your manuscript), or elaborate on this item by providing additional information not in the ms, or briefly explain why the item is not applicable/relevant for your study

Yes, Registration number and name of trial registry are listed like this in the abstract: "The trial is registered under the ClinicalTrials.gov number, NCT03412461"

24) Where the full trial protocol can be accessed, if available

Does your paper address CONSORT subitem 24? *

Cite a Multimedia Appendix, other reference, or copy and paste relevant sections from the manuscript (include quotes in quotation marks "like this" to indicate direct quotes from your manuscript), or elaborate on this item by providing additional information not in the ms, or briefly explain why the item is not applicable/relevant for your study

Yes, Full trial protocol and changes are included in Multimedia Appendix 1

25) Sources of funding and other support (such as supply of drugs), role of funders

Does your paper address CONSORT subitem 25? *

Copy and paste relevant sections from the manuscript (include quotes in quotation marks "like this" to indicate direct quotes from your manuscript), or elaborate on this item by providing additional information not in the ms, or briefly explain why the item is not applicable/relevant for your study

The sources of funding and other supports are described: "This project was supported by eHealth Innovation Partnership Program (eHIPP) grant from the Canadian Institute for Health Research (EH1- 143558). The Canadian Institute for Health Research was not involved in the study design, data analysis, data interpretation, and writing of the report."

X27) Conflicts of Interest (not a CONSORT item)

X27-i) State the relation of the study team towards the system being evaluated

In addition to the usual declaration of interests (financial or otherwise), also state the relation of the study team towards the system being evaluated, i.e., state if the authors/evaluators are distinct from or identical with the developers/sponsors of the intervention.

	1	2	3	4	5	
subitem not at all important	○	○	●	○	○	essential

Does your paper address subitem X27-i?

Copy and paste relevant sections from the manuscript (include quotes in quotation marks "like this" to indicate direct quotes from your manuscript), or elaborate on this item by providing additional information not in the ms, or briefly explain why the item is not applicable/relevant for your study

The relation of the study team towards the system being evaluated is stated like this:

"Applying principles from participatory action and participatory design research,[25, 26] the research team produced Thought Spot, a mHealth intervention co-created with transition-aged youth that aims to improve help-seeking behaviour related to mental health services for postsecondary settings.[27, 28] "

About the CONSORT EHEALTH checklist

As a result of using this checklist, did you make changes in your manuscript? *

- ○ yes, major changes
- ◉ yes, minor changes
- ○ no

What were the most important changes you made as a result of using this checklist?

Your answer

How much time did you spend on going through the checklist INCLUDING making changes in your manuscript *

Your answer

As a result of using this checklist, do you think your manuscript has improved? *

- (x) yes
- ( ) no
- ( ) Other:

Would you like to become involved in the CONSORT EHEALTH group?

This would involve for example becoming involved in participating in a workshop and writing an "Explanation and Elaboration" document

- ( ) yes
- ( ) no
- ( ) Other:

Any other comments or questions on CONSORT EHEALTH

Your answer

STOP - Save this form as PDF before you click submit

To generate a record that you filled in this form, we recommend to generate a PDF of this page (on a Mac, simply select "print" and then select "print as PDF") before you submit it.

When you submit your (revised) paper to JMIR, please upload the PDF as supplementary file.

Don't worry if some text in the textboxes is cut off, as we still have the complete information in our database. Thank you!

Final step: Click submit !

Click submit so we have your answers in our database!

Submit

Never submit passwords through Google Forms.

This content is neither created nor endorsed by Google. Report Abuse - Terms of Service - Privacy Policy

Google Forms
